# Identification and Comparative Analysis of the Peptidyl-Prolyl *cis/trans* Isomerase Repertoires of *H. sapiens, D. melanogaster, C. elegans, S. cerevisiae and Sz. pombe*

**DOI:** 10.1002/cfg.482

**Published:** 2005

**Authors:** Trevor J. Pemberton, John E. Kay

**Affiliations:** 1 The Brighton and Sussex Medical School University of Sussex Falmer Brighton East Sussex BN1 9PX United Kingdom; 2 Institute for Genetic Medicine Keck School of Medicine University of Southern California 2250 Alcazar Street, CSC-240 Los Angeles CA 90033 USA

## Abstract

The peptidyl-prolyl *cis/trans* isomerase (PPIase) class of proteins comprises three member families that are found throughout nature and are present in all the major compartments of the cell. Their numbers appear to be linked to the number of genes in their respective genomes, although we have found the human repertoire to be smaller than expected due to a reduced cyclophilin repertoire. We show here that whilst the members of the cyclophilin family (which are predominantly found in the nucleus and cytoplasm) and the parvulin family (which are predominantly nuclear) are largely conserved between different repertoires, the FKBPs (which are predominantly found in the cytoplasm and endoplasmic reticulum) are not. It therefore appears that the cyclophilins and parvulins have evolved to perform conserved functions, while the FKBPs have evolved to fill ever-changing niches within the constantly evolving organisms. Many orthologous subgroups within the different PPIase families appear to have evolved from a distinct common ancestor, whereas others, such as the mitochondrial cyclophilins, appear to have evolved independently of one another. We have also identified a novel parvulin within *Drosophila melanogaster* that is unique to the fruit fly, indicating a recent evolutionary emergence. Interestingly, the fission yeast repertoire, which contains no unique cyclophilins and parvulins, shares no PPIases solely with the budding yeast but it does share a majority with the higher eukaryotes in this study, unlike the budding yeast. It therefore appears that, in comparison with *Schizosaccharomyces pombe, Saccharomyces cerevisiae* is a poor representation of the higher eukaryotes for the study of PPIases.


**Comparative and Functional Genomics**
*Comp Funct Genom* 2005; **6**: 277–300.
Published online 11 July 2005 in Wiley InterScience (www.interscience.wiley.com). **DOI:** 10.1002/cfg.482

**Research Article**

### Identification and comparative analysis of the peptidyl-prolyl *cis/trans* isomerase repertoires of *H. sapiens, D. melanogaster, C. elegans, S. cerevisiae and Sz. pombe*

Trevor J. Pemberton* and John E. Kay
*The Brighton and Sussex Medical School, University of Sussex, Falmer, Brighton, East Sussex BN1 9PX, UK*

**Correspondence to:*
*Trevor J. Pemberton, Institute for Genetic Medicine, Keck School of Medicine, University of Southern California, 2250 Alcazar Street, CSC-240, Los Angeles, CA 90033, USA.*
*E-mail: trevorp@usc.edu*

Received: 31 January 2005
Revised: 1 May 2005
Accepted: 26 May 2005

### Abstract

**The peptidyl-prolyl *cis/trans* isomerase (PPIase) class of proteins comprises three member families that are found throughout nature and are present in all the major compartments of the cell. Their numbers appear to be linked to the number of genes in their respective genomes, although we have found the human repertoire to be smaller than expected due to a reduced cyclophilin repertoire. We show here that whilst the members of the cyclophilin family (which are predominantly found in the nucleus and cytoplasm) and the parvulin family (which are predominantly nuclear) are largely conserved between different repertoires, the FKBPs (which are predominantly found in the cytoplasm and endoplasmic reticulum) are not. It therefore appears that the cyclophilins and parvulins have evolved to perform conserved functions, while the FKBPs have evolved to fill ever-changing niches within the constantly evolving organisms. Many orthologous subgroups within the different PPIase families appear to have evolved from a distinct common ancestor, whereas others, such as the mitochondrial cyclophilins, appear to have evolved independently of one another. We have also identified a novel parvulin within *Drosophila melanogaster* that is unique to the fruit fly, indicating a recent evolutionary emergence. Interestingly, the fission yeast repertoire, which contains no unique cyclophilins and parvulins, shares no PPIases solely with the budding yeast but it does share a majority with the higher eukaryotes in this study, unlike the budding yeast. It therefore appears that, in comparison with *Schizosaccharomyces pombe, Saccharomyces cerevisiae* is a poor representation of the higher eukaryotes for the study of PPIases.** **Copyright © 2005 John Wiley & Sons, Ltd.**

**Keywords:** **peptidyl-prolyl *cis/trans* isomerase; cyclophilin; FKBP; parvulin**

### Introduction

The peptidyl-prolyl *cis/trans* isomerase (PPIase) class of protein is comprised of three known protein families, the cyclophilins (cyclosporin A binding proteins), FKBPs (FK506-binding proteins) and parvulins. These structurally distinct families are linked by their shared ability to catalyse the bond preceding a proline residue between its *cis* and *trans* forms. While they are all believed to employ the 'twisted amide' mechanism of catalysis, as is seen with prolyl isomerization in water, the cyclophilins (Eisenmesser *et al.*, 2002; Hur and Bruice, 2002) and parvulins (Ranganathan *et al.*, 1997) achieve this through the use of near attack conformers, whereas the FKBPs use hydrophobic distortion (Harrison and Stein, 1992; Hur and Bruice, 2002). They are found widely distributed in eukaryotes, prokaryotes and archaea (Galat, 1993, 1999; Galat and Metcalfe, 1995; He *et al.*, 2004; Ivery, 2000; Maruyama and Furuani, 2000; Rulten *et al.*, 1999), implying that their function is

Copyright © 2005 John Wiley & Sons, Ltd.

required in cellular processes from bacteria to man, and in all the major compartments of the cell (Bose *et al.*, 1994; Halestrap and Davidson, 1990; Handschumacher *et al.*, 1984; Jin and Burakoff, 1993; Lu *et al.*, 1996; Nigam *et al.*, 1993; Siekierka *et al.*, 1989; Uchida *et al.*, 1999; Wang *et al.*, 1996).

All of the cyclophilins and FKBPs in the budding yeast *Saccharomyces cerevisiae* have been individually and collectively knocked out with no effect on cell viability (Dolinski *et al.*, 1997a; Hemenway and Heitman, 1993). Only Ess1, the *S. cerevisiae* orthologue of the human parvulin Pin1, has been shown to be essential within *S. cerevisiae* (Hanes *et al.*, 1989), as has the Pin1 orthologue in the pathogenic yeast *Candida albicans* (Devasahayam *et al.*, 2002). However, the Pin1 orthologues in their fellow yeast *Schizosaccharomyces pombe* (Huang *et al.*, 2001), the fruit fly *Drosophila melanogaster* (Maleszka *et al.*, 1996) and *Cryptococcus neoformans* (Ren *et al.*, 2005) have been shown to be non-essential, indicating that the essential function of Pin1 orthologues is limited to certain organisms or that redundancy mechanisms are present in these other organisms. In the eubacterium *Bacillus subtilis*, the two cytosolic PPIases, PpiB and trigger factor, have been shown to be necessary for cell viability under starvation conditions (Gothel *et al.*, 1998) but they appear not to possess an essential function within a cell under normal growth conditions. In mammals, an FKBP12 knock-out mouse showed normal skeletal muscle but suffered from severe cardiomyopathy and ventricular septal defects that mimic a human congenital heart disorder (Shou *et al.*, 1998), an effect assigned to its modulation of calcium release activity of both skeletal and cardiac ryanodine receptors. Recently a mutation of the *D. melanogaster* cyclophilin CG3511, which severely truncates the protein, has been shown to confer a synthetic lethal phenotype on cells that lack the retinoblastoma (Rbf) protein (Edgar *et al.*, 2005). Despite the high conservation of the PPIases throughout the eukaryotes and prokaryotes, it appears that they do not possess an essential function within many cells under normal growth conditions but may become essential in the absence of other cellular factors.

Much of the research on the PPIases has been on individual proteins spread throughout many different organisms. Some recent reviews have considered different families of PPIases individually (Galat, 1999, 2004; Patterson *et al.*, 2002) but none have considered PPIases repertoires on a whole. We report here the comparative analysis of the PPIase repertoires of the mammal *Homo sapiens*, the fruit fly *D. melanogaster*, the nematode *Caenorhabditis elegans* and the two yeasts *Sz. pombe* and *S. cerevisiae*. By comparing these five diverse repertoires we hope to identify key conserved PPIases that are found within all their repertoires as well as those that are specific to each. By comparing the identified functions of each PPIase and its orthologues, we hope to understand better their functions within the cell and to identify those that function within a broad range of eukaryotes from those that are specific to multicellular eukaryotes.

### Materials and methods

### BLAST searching

The identification of putative PPIases and their orthology between repertoires was performed using both BLASTP (protein vs. protein) and TBLASTN (protein vs. DNA sequence) searches of the complete annotated genome sequences of *H. sapiens* (Lander *et al.*, 2001), *D. melanogaster* (Adams, 2000), *C. elegans* (The *C. elegans* Genome Consortium, 1998), *Sz. pombe* (Wood *et al.*, 2002) and *S. cerevisiae* (Goffeau *et al.*, 1996; Wood *et al.*, 2001) maintained by either the National Center for Biotechnology Information (NCBI) (Altschul *et al.*, 1997) (**http://www.ncbi.nlm.nih.gov/BLAST/**) or The Institute for Genomic Research (TIGR) (Gish *et al.*, 1990) (**http://tigrblast.tigr.org/tgi/**).

### Identification of the PPIase repertoires

The peptidyl-prolyl *cis/trans* isomerase (PPIase) repertoires present in the complete annotated genome sequences of *H. sapiens* (Lander *et al.*, 2001), *D. melanogaster* (Adams, 2000), *C. elegans* (The *C. elegans* Genome Consortium, 1998), *Sz. pombe* (Wood *et al.*, 2002) and *S. cerevisiae* (Goffeau *et al.*, 1996; Wood *et al.*, 2001) were identified using the protein sequences of human cyclophilin A (hCypA; Accession No. P05092), human FKBP12 (hFKBP12; P20071) and human Pin1 (hPin1; Q13526) as probes in BLASTP and TBLASTN searches of their sequences. Proteins were selected based upon the level of homology, both in regard to actual sequence homology and/or the presence of characteristic motifs, their PPIase

Copyright © 2005 John Wiley & Sons, Ltd. 

catalytic domain exhibited towards that of their probes sequence.

### Protein sequence analysis

The identification of putative domains within the identified PPIases was performed using two NCBI search engines, the CDD (Conserved Domain Database) world-wide web-based BLAST server (Altschul *et al*., 1997) (**http://www.ncbi.nlm.nih.gov/Structure/cdd/wrpsb.cgi**) and their CDART (Geer *et al*., 2002) (Conserved Domain Architecture Retrieval Tool; **http://www.ncbi.nlm.nih.gov/Structure/lexington/lexington.cgi**) analysis program.

The predicted localization of the PPIases and the identification of sequence motifs that support this were identified using the PSORT world-wide web-based search program (Horton and Nakai, 1996, 1997) located on the National Institute for Basic Biology (NIBB) server (**http://psort.nibb.ac.jp/**). The theoretical molecular weights of the predicted proteins were calculated using the calculation tool on the ExPaSy server (**http://www.expasy.ch/tools/pi_tool.html**).

### Comparative sequence analysis

Alignments of each family of PPIases were produced using version 1.81 of the ClustalX program (Thompson *et al*., 1997) downloaded from **http://inn-prot.weizmann.ac.il/software/ClustalX.html**. This program performs a pairwise alignment of the sequences prior to the construction of a dendrogram, which describes the approximate groupings of the sequences by similarity, with the final alignment carried out using this dendrogram as a guide. The dendrogram was visualized using TreeView version 1.6.6 (Dr R Page; University of Glamorgan) downloaded from **http://taxonomy.zoology.gla.ac.uk/rod/rod.html** from the files generated by the ClustalX alignment. The scales of the different dendrograms are not cross-comparable.

### Identification of orthologues

PPIases were considered to be orthologues if they fulfilled three criteria. First, they should be of approximately the same size and possess the same domain architecture. Second, in BLAST searches they should identify each other ahead of all other PPIases within their respective genomes. This is because they should, in theory, share a more recent common ancestor than they do with the other PPIases. Sequence variation, resulting from the distinct divergent evolution of each protein, should therefore be less between two orthologues than with other PPIases. Third, they should have the same intracellular location and function. This latter criterion is, however, reliant upon prior research, which is not applicable to all PPIases. In these cases, so long as the first two criteria were met, the proteins were deemed to be putative orthologues.

Three methods were employed to identify the orthology between the repertoires and answer the above criteria. First, the individual PPIases were used as probes in BLAST searches of the other species' genomes. Second, the sequences for all the member proteins from all of the comparative organisms of each of the three different PPIase families (cyclophilins, FKBPs and parvulins) were subjected to global sequence comparison by family, using the ClustalX program (Thompson *et al*., 1997) for the purpose of creating a dendrogram. This analysis creates a putative model for how the individual subgroups of each PPIase family may have diverged from one another, based on relationships between their individual sequences, which allow us to infer a putative model for their evolution in the species compared here. As each orthology group should share a more recent common ancestor with themselves than with the other PPIases, they should group together within the dendrogram, ideally as an individual branch with a distinct common ancestor. Third, literature analysis looking for prior publications on the individual PPIases was performed, which in some cases has allowed putative function(s) to be assigned to orthology groups.

### Results

The identified PPIase repertoires of *H. sapiens, D. melanogaster, C. elegans, Sz. pombe* and *S. cerevisiae* can be found in Tables 1 and 2. Table 3 shows a comparison of the number of members of each PPIase family found within the different species. The repertoire orthology of the cyclophilins, FKBPs and parvulins as identified by BLAST analysis can be found in Table 4.

Copyright © 2005 John Wiley & Sons, Ltd. 

**Table 1.** Peptidyl-prolyl *cis/trans* isomerases identified by BLAST searching of the NCBI database of the complete genome and proteome of the multicellular organisms (A) *H. sapiens* (Lander *et al.*, 2001), (B) *D. melanogaster* (Adams, 2000) and (C) *C. elegans* (The C. elegans Genome Consortium, 1998). Localization was predicted using the PSORT II server, molecular weight was predicted using the ExPaSy server and domains were identified using the CCD BLAST program on the NCBI server apart from the *Moca* domain, which was included for those cyclophilins identified as possessing one by Cavarec *et al.* (2002)

| A | PPlase | Uniprot Acc. # | kDa | Signal Seq. | Predicted Localisation | Domain Architecture |
|---|---|---|---|---|---|---|
| Cyclophilins | Cyp-A | P62937 | 18.0 | — | Cytoplasmic | PPlase Only |
| | PPIL1 (CGI-124) | Q9Y3C6 | 18.2 | ~ | Cytoplasmic | PPlase Only |
| | PPIL3 | Q9BXZ1 | 18.6 | ~ | Cytoplasmic | PPlase Only |
| | USA-CyP (Cyp-H) | O43447 | 19.2 | — | Cytoplasmic | PPlase Only |
| | Cyp-F (D) | P30405 | 22.0 | — | Mitochondrial | PPlase Only |
| | Cyp-B | Q9BVK5 | 22.7 | N-term | ER | PPlase Only |
| | Cyp-C | P45877 | 22.8 | N-term | ER | PPlase Only |
| | "Cyp29" | P49069 | 28.9 | ~ | Nuclear | N-term PPlase Only |
| | Cyp33 (E) | Q9UNP9 | 33.4 | — | Cytoplasmic | N-term RRM, C-term PPlase |
| | Cyp40 (D) | Q08752 | 40.8 | — | Cytoplasmic | N-term PPlase, C-term TPR (3x) motifs |
| | SDCCAG10 | Q6UX04 | 53.8 | — | Nuclear | N-term PPlase, positively charged C-term |
| | "Cyp57" | Q8WUA2 | 57.2 | ~ | Nuclear | N-term PPlase, Central RRM |
| | Cyp60 | Q13356 | 58.8 | ~ | Nuclear | N-term U-box, C-term PPlase |
| | HAL539 | Q96BP3 | 73.6 | — | Cytoplasmic | N-term WD40 (×3) motifs, C-term PPlase |
| | Cyp88 (CARS/G) | Q13427 | 88.6 | — | Nuclear | N-term PPlase, C-term RS domain |
| | NK-Cyp (158/SR) | P30414 | 165.7 | — | Nuclear | N-term PPlase C-term RS domain containing a *Moca* motif |
| | RanBP2 | P49792 | 358.2 | — | Nuclear | N-term TPR, central Zn-fingers, central/C-term RB1 domains, C-term PPlase |
| FKBPs | FKBP12.6 | P68106 | 11.8 | — | Cytoplasmic | FKBP (×1) Only |
| | FKBP12 | P62942 | 12.0 | — | Cytoplasmic | FKBP (×1) Only |
| | FKBP13 | P26885 | 15.6 | N-term | Mitochondrial | FKBP (×1) Only |
| | FKBP19 | Q9NYL4 | 22.2 | N-term | ER | FKBP (×1) Only |
| | FKBP22 | Q9NWM8 | 24.3 | N-term | ER | FKBP (×1) Only |
| | FKBP23 | Q9Y680 | 30.0 | — | ER | N-term FKBP (×1), C-term EF-Hand motif |
| | FKBP25 | Q00688 | 25.2 | — | Cytoplasmic | C-term FKBP (×1) Only |
| | FKBP36 | O75344 | 37.2 | ~ | Cytoplasmic | N-term FKBP (×1), C-term TPR (×2) motifs |
| | FKBP38 | Q14318 | 38.4 | — | Cytoplasmic | N-term FKBP (×1), C-term TPR (×2) motifs |
| | FKBP51 | Q13451 | 51.2 | — | Cytoplasmic | N-term FKBP (×2), C-term TPR (×2) motifs |
| | FKBP52 | Q02790 | 51.6 | — | Cytoplasmic | N-term FKBP (×2), C-term TPR (×3) motifs |
| | FKBP60 | O95302 | 57.2 | ~ | ER | N-term FKBP (×4), C-term EF-Hand motif |
| | FKBP65 | Q96AY3 | 64.7 | N-term | ER | N-term FKBP (×4), C-term EF-Hand motif |
| Parvs | Pin1 | Q13526 | 9.0 | — | Nuclear | N-term WW domain, C-term Rotamase |
| | Par14 | Q9Y237 | 13.9 | — | Cytoplasmic | Rotamase Only |

Figure 1 shows the dendrograms generated for the cyclophilins, FKBPs and parvulins.

### Cyclophilin orthology

Table 4D shows that *D. melanogaster* and *H. sapiens* share the greatest number of orthologues, closely followed by their orthology to *C. elegans*. Of the two yeasts, *Sz. pombe* shares its entire repertoire of nine in common with *D. melanogaster* and *H. sapiens*, compared to only three shared by *S. cerevisiae*, which has a potential fourth in common (ScCwc27) that is discussed below. *Sz. pombe* is therefore the only organism in this comparison that has no unique cyclophilins within its repertoire and, interestingly, its repertoire shows less orthology to that of its fellow yeast *S. cerevisiae* than to the higher eukaryotes.

Table 4A shows that there are only two cyclophilin groups found in all five of the organisms. One group are the cyclophilin A orthologues, a ubiquitous group of cyclophilins that have been identified within the cytoplasm (Handschumacher *et al.*, 1984; Harding *et al.*, 1986; Huh *et al.*, 2003), although a recent report has found ScCpr1

Copyright © 2005 John Wiley & Sons, Ltd.

**Table 1.** Continued

| B | PPlase | Uniprot Acc. # | kDa | Signal Seq. | Predicted Localisation | Domain Architecture |
|---|---|---|---|---|---|---|
| Cyclophilins | CG7768 | Q9VUD6 | 17.8 | — | Cytoplasmic | PPlase Only |
| | CG11777 | Q8MKJ6 | 17.8 | — | Cytoplasmic | PPlase Only |
| | Cyp1 | P25007 | 17.9 | ~ | Cytoplasmic | PPlase Only |
| | CG13892 | Q9W0Q2 | 19.5 | ~ | Cytoplasmic | PPlase Only |
| | CG17266 | Q9V9B9 | 20.2 | — | Cytoplasmic | PPlase Only |
| | CG2852 | Q9W227 | 22.2 | N-term | ER | PPlase Only |
| | NinaA | P15425 | 26.4 | N-term | ER | PPlase Only |
| | Cyp-33 | Q9V3G3 | 33.3 | ~ | Cytoplasmic | N-term RRM, C-term PPlase |
| | CG8336 | Q9VT21 | 43.1 | ~ | Cytoplasmic | N-term PPlase, C-term TPR (2×) motifs |
| | CG10907 | Q9VTN7 | 56.6 | — | Nuclear | N-term PPlase, C-term RS domain |
| | CG7747 | Q9V7M9 | 59.0 | — | Nuclear | N-term U-box, C-term PPlase |
| | CG3511 | Q960Q8 | 71.8 | ~ | Cytoplasmic | N-term WD40 motif, C-term PPlase |
| | CG5808 | Q9XYZ6 | 75.5 | ~ | Nuclear | N-term PPlase, central RRM, C-term RS domain |
| | Moca-Cyp | Q8ISE5 | 112.5 | — | Nuclear | N-term PPlase C-term RS domain containing a *Moca* motif |
| FKBPs | FKBP12 | P48375 | 11.6 | ~ | Cytoplasmic | FKBP (1×) Only |
| | CG14715 | Q9VGK3 | 14.8 | N-term | ER | FKBP (1×) Only |
| | FKBP13 | Q8MLW1 | 25.7 | — | ER | Central FKBP (1×), C-term EF-Hand motif |
| | FKBP39 | P54397 | 39.3 | — | Nuclear | Positively charged N-term, C-term FKBP (1×) |
| | CG5482 | Q9V8K4 | 44.9 | — | Cytoplasmic | N-term FKBP (1×), C-term TPR (2×) motifs |
| | FKBP59 | Q9VL78 | 48.8 | ~ | Cytoplasmic | N-term FKBP (2×), C-term TPR (2×) motifs |
| | shutdown | Q9W1I9 | 51.8 | ~ | Nuclear | Central FKBP (1×), C-term TPR (1×) motif |
| Parvulins | Dodo | P54353 | 18.4 | — | Nuclear | N-term WW domain, C-term Rotamase |
| | CG11858 | Q9VBU4 | 13.9 | ~ | Cytoplasmic | Rotamase Only |
| | CG32845 | Q8IRJ5 | 44.3 | ~ | Nuclear | Central Rotamase Only |

| C | PPlase | Uniprot Acc. # | kDa | Signal Seq. | Predicted Localisation | Domain Architecture |
|---|---|---|---|---|---|---|
| Cyclophilins | Cyp1 | P52009 | 20.7 | ~ | Mitochondrial | PPlase Only |
| | Cyp2 | P52010 | 18.5 | ~ | Cytoplasmic | PPlase Only |
| | Cyp3 | P52011 | 18.6 | ~ | Cytoplasmic | PPlase Only |
| | Cyp4 | P52012 | 58.5 | — | Cytoplasmic | N-term U-box, C-term PPlase |
| | Cyp5 | P52013 | 22.4 | N-term | ER | PPlase Only |
| | Cyp6 | P52014 | 21.9 | N-term | ER | PPlase Only |
| | Cyp7 | P52015 | 18.4 | ~ | Cytoplasmic | PPlase Only |
| | Cyp8 | P52016 | 53.6 | — | Nuclear | N-term PPlase C-term RS domain containing a *Moca* motif |
| | Cyp9 | Q09637 | 35.8 | ~ | Nuclear | N-term PPlase C-term RS domain containing a *Moca* motif |
| | Cyp10 | P52017 | 18.0 | — | Cytoplasmic | PPlase Only |
| | Cyp11 | P52018 | 20.2 | ~ | Cytoplasmic | PPlase Only |
| | Cyp12 | Q18445 | 18.5 | ~ | Cytoplasmic | PPlase Only |
| | Cyp13 | Q9U2S6 | 36.4 | — | Cytoplasmic | N-term RRM, C-term PPlase |
| | Cyp14 | O18161 | 50.4 | — | Nuclear | N-term PPlase, C-term RRM |
| | Cyp15 | Q9U1Q3 | 70.8 | — | Cytoplasmic | N-term WD40 motifs, C-term PPlase |
| | Cyp16 | Q9XX17 | 25.2 | ~ | Cytoplasmic | PPlase Only |
| | Cyp17 | O01880 | 57.7 | ~ | Nuclear | Positively charged N-term, C-term PPlase |
| FKBPs | Fkb1 | Q20107 | 15.5 | N-term | Cytoplasmic | FKBP (1×) Only |
| | Fkb2 | Q9U2Q8 | 11.6 | ~ | Cytoplasmic | FKBP (1×) Only |
| | Fkb3 | O16309 | 29.1 | N-term | ER | FKBP (2×) Only |
| | Fkb4 | Q23338 | 29.3 | N-term | ER | FKBP (2×) Only |
| | Fkb5a | P91180 | 29.9 | N-term | ER | FKBP (2×) Only |
| | Fkb6 | O45418 | 48.1 | ~ | Cytoplasmic | N-term FKBP (2×), C-term TPR (3×) motifs |
| | Fkb7 | O61826 | 36.2 | N-term | ER | N-term FKBP (1×), C-term EF-hand motif |
| | Fkb8 | Q8I4L5 | 32.4 | ~ | Cytoplasmic | FKBP (2×) Only |
| Parvs | Pin1 | Q9N492 | 19.2 | — | Nuclear | N-term WW domain, C-term Rotamase |
| | Pin2 | Q9NAF9 | 13.3 | — | Nuclear | Rotamase Only |

Copyright © 2005 John Wiley & Sons, Ltd.

**Table 2.** Peptidyl-prolyl *cis/trans* isomerases identified by BLAST searching of the NCBI database of the complete genome and proteome of the unicellular organisms (A) *Sz. pombe* (Wood *et al.*, 2002) and (B) *S. cerevisiae* (Goffeau *et al.*, 1996). Localization was predicted using the PSORT II server, molecular weight was predicted using the ExPaSy server and domains were identified using the CCD BLAST program on the NCBI server

| A | PPlase | Uniprot Acc. # | kDa | Signal Seq. | Predicted Localisation | Domain Architecture |
|---|---|---|---|---|---|---|
| Cyclophilins | Cyp1 | P87051 | 17.4 | — | Cytoplasmic | PPlase only |
| | Cyp2 | P18253 | 16.9 | ~ | Cytoplasmic | PPlase only |
| | Cyp3 | O74729 | 18.9 | ~ | Cytoplasmic | PPlase only |
| | Cyp4 | O94273 | 22.2 | N-term | ER | PPlase only |
| | Cyp5 | Q11004 | 40.2 | — | Cytoplasmic | N-term. PPlase, C-term. TPR(×3) |
| | Cyp6 | Q9UUE4 | 50.8 | — | Nuclear | N-term. PPlase, C-term. RRM |
| | Cyp7 | O42941 | 52.2 | — | Nuclear | N-term. PPlase, C-term. positively charged |
| | Cyp8 | Q09928 | 53.6 | ~ | Nuclear | N-term. U-Box, C-term. PPlase |
| | Cyp9 | O74942 | 69.0 | ~ | Cytoplasmic | N-term. WD40(×3), C-term. PPlase |
| FKBPs | FKBP12 | O42993 | 12.0 | — | Cytoplasmic | FKBP only |
| | FKBP39 | O74191 | 39.3 | — | Nuclear | N-term. Positively charged, C-term. FKBP |
| | FKBP39a | Q10175 | 40.5 | — | Cytoplasmic | N-term. Positively charged, C-term. FKBP |
| Parv | Pin1 | O74448 | 19.8 | ~ | Nuclear | N-term. WW domain, C-term. Rotamase |
| **B** | **PPlase** | **Uniprot Acc. #** | **kDa** | **Signal Seq.** | **Predicted Localisation** | **Domain Architecture** |
| Cyclophilins | Cpr1 | P14832 | 17.4 | ~ | Cytoplasmic | PPlase Only |
| | Cpr2 | P23285 | 22.8 | N-term | ER | PPlase Only |
| | Cpr3 | P25719 | 19.9 | — | Mitochondrial | PPlase Only |
| | Cpr4 | P25334 | 35.8 | N-term | ER | Central PPlase Only |
| | Cpr5 | P35176 | 25.3 | N-term | ER | PPlase Only |
| | Cpr6 | P53691 | 42.1 | — | Cytoplasmic | N-term PPlase, C-term TPR(3×) motifs |
| | Cpr7 | P47103 | 45.1 | — | Cytoplasmic | N-term PPlase, C-term TPR (3×) motifs |
| | Cpr8 | P53728 | 34.9 | — | Membrane | Central PPlase |
| | *Cwc27* | *Q02770* | *35.0* | ~ | *Nuclear* | *Divergent N-term PPlase Only* |
| FKBPs | Fpr1 | P20081 | 12.2 | ~ | Cytoplasmic | FKBP (1×) Only |
| | Fpr2 | P32472 | 14.5 | N-term | ER | FKBP (1×) Only |
| | Fpr3 | P38911 | 46.6 | — | Nuclear | Negatively charged N-term, C-term FKBP (1×) |
| | Fpr4 | Q06205 | 43.9 | — | Nuclear | Negatively charged N-term, C-term FKBP (1×) |
| Parv | ESS1 | P22696 | 21.7 | — | Nuclear | N-term WW domain. C-term Rotamase |

to be nuclear (Arevalo-Rodriguez and Heitman, 2005), which is contrary to that found by Huh *et al.* (Huh *et al.*, 2003). Members of this group have been reported to function in protein folding (Davis *et al.*, 1989; Kern *et al.*, 1995; Reader *et al.*, 2001; Steinmann *et al.*, 1991), protein activity regulation (Ansari *et al.*, 2002; Brazin *et al.*, 2002; Yurchenko *et al.*, 2005), transcriptional regulation (Ansari *et al.*, 2002; Arevalo-Rodriguez *et al.*, 2000; Pijnappel *et al.*, 2001), receptor signalling pathways (Allain *et al.*, 1994; Bukrinsky, 2002; Syed, 2003; Huang *et al.*, 2002; Nagata *et al.*, 2000; Pushkarsky *et al.*, 2001; Rycyzyn *et al.*, 2000; Yurchenko *et al.*, 2001, 2002), apoptosis (Cande *et al.*, 2004), the cellular oxidative stress response (Jin *et al.*, 2000; Lee *et al.*, 2001), a vesicular import pathway (Brown *et al.*, 2001) and in the control of both the meiotic (Arevalo-Rodriguez and Heitman, 2005) and mitotic cell cycles in *S. cerevisiae* (Fujimori *et al.*, 2001).

The other group are the cyclophilin B orthologues, a group identified by their targeting to the endoplasmic reticulum (ER) (Frigerio and Pelham, 1993; Kumar *et al.*, 2002; Price *et al.*, 1991) courtesy of their N-terminal signal peptide. They function within the secretory pathway, where they have been reported to be involved in the chaperoning of plasma membrane proteins (Horibe *et al.*,

Copyright © 2005 John Wiley & Sons, Ltd. 

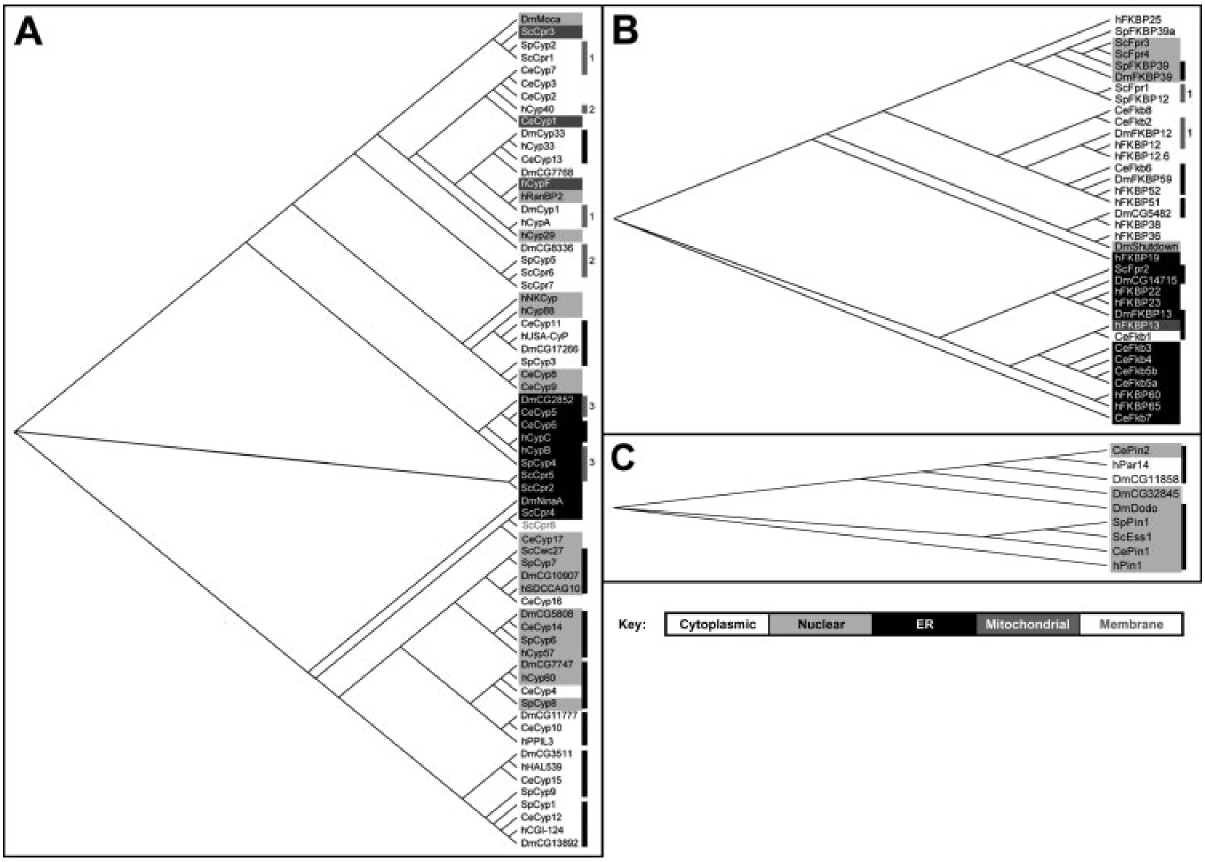

**Figure 1.** Dendrograms depicting the predicted history of divergence of (A) the cyclophilins, (B) the FKBPs and (C) the parvulins of *Sz. pombe* (Sp), *S. cerevisiae* (Sc), *D. melanogaster* (Dm), *C. elegans* (Ce) and *H. sapiens* (h), based upon a comparison of their protein sequences by the ClustalX program (Thompson *et al.*, 1997) with the dendrogram generated using TreeView version 1.6.6 (Dr R Page; University of Glamorgan). Background and text colours identify their PSORT predicted localization. Bars up the right hand side indicate groups identified as orthologous by BLAST analysis (Table 4). Black bars indicate a group where all members are found at the same point in the dendrogram. Groups with members in more than one location are shown by grey bars and identified by the numbers to their right (key: (A) CypA, group 1; CypB, group 2; Cyp40, group 3; (B) FKBP12, group 1)

2002; Klappa *et al.*, 1995; Meunier *et al.*, 2002; Price *et al.*, 1991, 1994; Zhang and Herscovitz, 2003) and also have proposed functions in receptor signalling pathways (Allain *et al.*, 1994; Bukrinsky, 2002; Nagata *et al.*, 2000; Obata *et al.*, 2005; Rycyzyn and Clevenger, 2002; Rycyzyn *et al.*, 2000; Yurchenko *et al.*, 2001).

Only two other groups have an *S. cerevisiae* member but both lack an apparent *C. elegans* orthologue (Table 4A). The cyclophilin 40 orthologues are a group of heat shock-inducible (Lebeau *et al.*, 1999; Mark *et al.*, 2001; Mayr, 2000; Weisman *et al.*, 1996), predominantly nuclear (Huh *et al.*, 2003; Lebeau *et al.*, 1999; Mark *et al.*, 2001) cyclophilins that interact with the C-terminal MEEVD pentapeptide of heat shock protein 90 (Hsp90) (Ward *et al.*, 2002) and have been reported to function within the Hsp90 complex (Davies *et al.*, 2005; Duina *et al.*, 1998; Sykes *et al.*, 1993), potentially regulating its ATPase activity (Prodromou *et al.*, 1999) during its functions in cellular signalling pathways that regulate transcription (Pratt and Toft, 1997; Sanchez and Ning, 1996; Ward *et al.*, 2001; Warth *et al.*, 1997), the cellular heat shock response (Bharadwaj *et al.*, 1999) and also in maintaining the cell cycle protein kinases Mik1, Wee1 and Swe1 (Goes and Martin, 2001). Interestingly, the Hsp90 complex is

Copyright © 2005 John Wiley & Sons, Ltd. 

present within *C. elegans* (Birnby *et al*., 2000), making the absence of an associated cyclophilin surprising.

The second group contains both ScCwc27, which has not formed part of previous research on the *S. cerevisiae* repertoire due to the presence of a very degenerate PPIase domain, and hSDCCAG10, which was formerly called hNY-CO-10 until its re-annotation to include a complete cyclophilin-like catalytic domain. They share a region rich in S/K-R/E residues that is similar to those observed in hnRNP-binding proteins (Romano *et al*., 2004; Weighardt *et al*., 1999) (data not shown) and both

**Table 3.** The numbers of the three different families that make up the peptidyl-prolyl *cis/trans* isomerase repertoires of *H. sapiens, D. melanogaster, C. elegans, S. cerevisiae* and *Sz. pombe*

| Organism | Genes | Cyclo-philins | FKBPs | Par-vulins | Total |
|---|---|---|---|---|---|
| *H. sapiens* | 24-40.000[a] | 17 | 13 | 2 | 32 |
| *C. elegans* | 18.424[b] | 17 | 8 | 2 | 27 |
| *D. melanogaster* | 13.601[b] | 14 | 7 | 3 | 24 |
| *S. cerevisiae* | 5.885[c] | 8 | 4 | 1 | 13 |
| *Sz. pombe* | 4.824[d] | 9 | 3 | 1 | 13 |

[a] Lander *et al.* (2001); [b] Rubin *et al.* (2000); [c] Goffeau *et al.* (1996); [d] Wood *et al.* (2002).

**Table 4.** Orthology between the (A) cyclophilin, (B) FKBP and (C) parvulin repertoires of *S. cerevisiae, Sz. pombe, D. melanogaster, C. elegans* and *H. sapiens*, identified by BLAST searching of the NCBI database of their complete genomes and proteomes (Adams, 2000; The C. elegans Genome Consortium, 1998; Goffeau *et al*., 1996; Lander *et al*., 2001; Wood *et al*., 2002, respectively). PPIases are ordered by increasing size and PSORT predicted localization with any secondary domains or domain architecture shown down the left hand side (ND = none detected; ± = charged region). (D) The number of orthologues shared between the different repertoires. Cyclophilins (top) and FKBPs (bottom) are shown on the right with the parvulins shown on the left. *S. cerevisiae* numbers outside of brackets are exclusive of, and numbers within brackets are inclusive of, ScCwc27

**A**

| | *S. cerevisiae* | *Sz. pombe* | *D. melanogaster* | *C. elegans* | *H. Sapiens* |
|---|---|---|---|---|---|
| ND | Cpr1 | Cyp2 | Cyp1<br>CG7768 | Cyp7<br>Cyp2<br>Cyp3 | Cyp-A |
| | | Cyp1 | CG13892 | Cyp12 | CGI-124 (PPIL1) |
| | | | CG11777 | Cyp10 | PPIL3 |
| | | Cyp3 | CG17266 | Cyp11 | USA-CyP (Cyp-H) |
| | | | | Cyp16 | |
| RRM | | | Cyp33 | Cyp13 | Cyp33 (E) |
| TPR | Cpr6<br>Cpr7 | Cyp5 | CG8336 | | Cyp40 (D) |
| WD40 | | Cyp9 | CG3511 | Cyp15 | HAL539 |
| ND | | | | | "Cyp29" |
| RRM | | Cyp6 | CG5808 | Cyp14 | "Cyp57" |
| +/- | Cwc27 | Cyp7 | CG10907 | | SDCCAG10 |
| ND | | | | Cyp17 | |
| U-box | | Cyp8 | CG7747 | Cyp4 | Cyp60 |
| RS | | | | | Cyp88 (CARS/G) |
| RS & MOCA | | | | | NK-Cyp (158/SR) |
| | | | Moca-Cyp | | |
| | | | | Cyp8 | |
| | | | | Cyp9 | |
| TPR, RB1 & ZF | | | | | RanBP2 |
| ND | Cpr5 | Cyp4 | CG2852 | Cyp5 | Cyp-B |
| | | | | Cyp6 | Cyp-C |
| | Cpr2 | | | | |
| | | | NinaA | | |
| | Cpr4 | | | | |
| ND | | | | | Cyp-F (D) |
| | | | | Cyp1 | |
| | Cpr3 | | | | |
| ND | Cpr8 | | | | |

**B**

| | *S. cerevisiae* | *Sz. pombe* | *D. melanogaster* | *C. elegans* | *H. Sapiens* |
|---|---|---|---|---|---|
| 1xFKBP | Fpr1 | FKBP12 | FKBP12 | Fkb2 | FKBP12<br>FKBP12.6 |
| | | | | | FKBP25 |
| 2xFKBP | | | | Fkb8 | |
| 1xFKBP & 1xTPR | | | | | FKBP36 |
| | | | | | FKBP38 |
| 1xFKBP & +/- | | FKBP39a | | | |
| 2xFKBP & 1xTPR | | | CG5482 | | FKBP51 |
| | | | FKBP59 | Fkb6 | FKBP52 |
| 1xFKBP & +/- | Fpr3 | | | | |
| | Fpr4 | | | | |
| | | FKBP39 | FKBP39 | | |
| 1xFKBP & 1xTPR | | | shutdown | | |
| 1xFKBP | Fpr2 | | CG14715 | | |
| | | | | | FKBP19 |
| | | | | | FKBP22 |
| 1xFKBP & 1xEF | | | | | FKBP23 |
| | | | | Fkb7 | |
| 2xFKBP | | | | Fkb3 | |
| | | | | Fkb4 | |
| | | | | Fkb5 | |
| 4xFKBP & 1xEF | | | | | FKBP60 |
| | | | | | FKBP65 |
| 1xFKBP | | | FKBP13 | Fkb1 | FKBP13 |

**C**

| | *S. cerevisiae* | *Sz. pombe* | *D. melanogaster* | *C. elegans* | *H. Sapiens* |
|---|---|---|---|---|---|
| ND | | | CG11858 | Pin2 | Par14 |
| WW | ESS1 | Pin1 | Dodo | Pin1 | Pin1 |
| ND | | | CG32845 | | |

| Key: | Cytoplasmic | Nuclear | ER | Mitochondrial | Membrane |
|---|---|---|---|---|---|

**D**

| *S. cerevisiae* | *Sz. pombe* | *C. elegans* | *D. melanogaster* | *H. sapiens* | Species | *H. sapiens* | *D. melanogaster* | *C. elegans* | *Sz. pombe* | *S. cerevisiae* |
|---|---|---|---|---|---|---|---|---|---|---|
| 1 | 1 | 2 | 2 | | *H. sapiens* | | 11 | 10 | 9 | 3(4) |
| 1 | 1 | 2 | | | *D. melanogaster* | 4 | | 10 | 9 | 3(4) |
| 1 | 1 | | | | *C. elegans* | 3 | 3 | | 7 | 2 |
| 1 | | | | | *Sz. pombe* | 1 | 2 | 1 | | 3(4) |
| | | | | | *S. cerevisiae* | 1 | 2 | 1 | 1 | |

Copyright © 2005 John Wiley & Sons, Ltd.

SpCyp7 and ScCwc27 have been reported to be components of their respective Cdc5 complexes (Ohi *et al.*, 2002), but their function within this complex remains unknown. The apparent absence of a *C. elegans* orthologue cannot be explained by an absence of the Cdc5 complex, which has been reported as present within the nematode (Ohi *et al.*, 1998).

There are five groups that lack only an *S. cerevisiae* member (Table 4A), highlighting the large difference between the two yeast repertoires. Three groups are predicted to be cytoplasmic, of which one group has hCGI-124 as a member. hCGI-124 has been reported to be highly expressed in the heart and adult brain (Ozaki *et al.*, 1996) and members of this group have been identified as orthologues of *Dictyostelium discoideum* CypE (Skruzny *et al.*, 2001), which gives them a role within a broad range of signal transduction pathways (Skruzny *et al.*, 2001).

In the second group, hUSA-CyP (also called hCyp20) has been previously reported to be an orthologue of SpCyp3 (Pemberton *et al.*, 2003) and to associate with two components of the pre-mRNA spliceosome (Horowitz *et al.*, 1997), where it is required for the second stage of pre-mRNA splicing (Horowitz *et al.*, 2002). Both have been found to be predominantly nuclear (Pemberton *et al.*, 2003; Teigelkamp *et al.*, 1998), contrary to the PSORT prediction. The absence of nuclear localization sequences within their sequence (Pemberton *et al.*, 2003) would imply that functional interactions lead to their translocation to the nucleus. The role in pre-mRNA splicing would explain the absence of an *S. cerevisiae* orthologue, as the budding yeast performs very limited mRNA splicing in comparison with the other organisms.

The third cytoplasmic group has the human cyclophilin HAL539 as a member. Its members possess WD40 motifs in their N-terminal region, which are found in all eukaryotes, but not in prokaryotes, in a large variety of proteins that share no obvious commonality in their functions (Neer *et al.*, 1994). Recently, the severe truncation of DmCG3511 has been reported to cause synthetic lethality to cells lacking the Rbf protein (Edgar *et al.*, 2005), an orthologue of the human retinoblastoma protein that is linked to many human cancers, but it remains unknown by what mechanism this lethality is caused.

The remaining two orthology groups that lack an *S. cerevisiae* member are predicted to be nuclear (Table 4A). The group containing SpCyp6 and CeCyp14 all possess an RNA recognition motif (RRM), which is found in metazoan protein factors involved in constitutive pre-mRNA splicing and alternative splicing regulation (Birney *et al.*, 1993), and appear to be RNA-interacting cyclophilins linked to cell morphogenesis, cortical organization and nuclear reorganization (Krzywicka *et al.*, 2001).

The second is a predominantly nuclear group that contains hCyp60. Its members possess a U-Box motif, which is reported to be a modified RING-finger motif involved in protein–protein interactions that has been primarily identified in proteins involved in the ubiquitin–proteasome system (Pringa *et al.*, 2001). Expressed in the thymus, pancreas, testis and kidney, hCyp60 is a nuclear cyclophilin that interacts with the well-characterized leech serine-proteinase inhibitor elgin-c (Wang *et al.*, 1996). CeCyp4 has been found to be important in larval muscle development (Page and Winter, 1998) in the nematode, and interestingly is predicted by PSORT to be cytoplasmic, rather than nuclear like the rest of this group, which is also contrary to the reported localization of hCyp60 (Wang *et al.*, 1996).

The three remaining groups all lack a yeast member, containing members solely from the higher eukaryotes (Table 4A), indicating that their role is required solely within the additional pathways found within these multicellular organisms. Two of these groups are cytoplasmic, of which one contains hPPIL3 (Zhou *et al.*, 2001) and CeCyp10 (Page *et al.*, 1996), but as yet no functions have been identified for its members. Members of the second group possess an RRM and include the human RNA-binding cyclophilin hCyp33 (Mi *et al.*, 1996), DmCyp33, which has been reported to interact with the trx/MLL protein family which modulate the expression of the HOXC genes (Anderson *et al.*, 2002) and CeCyp13, which is found in an essential polycistronic operon (Mazroui *et al.*, 1999) but has itself been shown to be non-essential (Zorio and Blumenthal, 1999).

The final group is found within the endoplasmic reticulum and contains only two members; hCypC and CeCyp6 (Table 4A). hCypC is reported to function in the secretory pathway of specific tissues (Friedman *et al.*, 1993), namely bone marrow,

Copyright © 2005 John Wiley & Sons, Ltd. 

ovaries, testis and kidney (Friedman *et al*., 1994), and CeCyp6 was reported to localize exclusively to the nematode's gut (Picken *et al*., 2002).

The dendrogram shown in Figure 1A backs up many of the BLAST-identified orthology groupings detailed above. These groups are found clustered on the same distinct branch of the dendrogram leading back to their distinct common ancestor. Many of these branches show their members segregating in agreement with the divergence of the compared species (*Sz. pombe* and *S. cerevisiae*, followed by *C. elegans*, then *D. melanogaster* and finally *H. sapiens*). The groups with hCGI-124, hSDCCAG10 and hCyp60 would be good examples of this (Figure 1A, lower branch). The group containing human PPIL3 appears evolutionarily linked to the hCyp60 group and the group containing hHAL539 appears evolutionarily linked to the hCGI-124 group. The latter shows SpCyp9 separating from the other members of its group prior to their split with the hCGI-124 group. The others all segregate in order of species divergence from a distinct common ancestor, implying that SpCyp9 may be more distantly related to this group.

In the upper branch of the dendrogram (Figure 1A) only the hCyp33 group is found to diverge from a distinct common ancestor. The position of their branch within the dendrogram indicates that they are linked with the evolution of the cyclophilin As. The latter are seen to appear within the same branch of the dendrogram but without a common ancestor that is distinct only to them. They do, however, follow the same order of divergence as the species they are a part of, with both SpCyp2 and ScCpr1 diverging first, followed by CeCyp7 and then finally DmCyp1 and hCypA.

As was seen with the hHAL539 group, the hUSA-CyP group shows SpCyp3 to diverge away from the others prior to the distinct common ancestor from which all the others diverged (Figure 1A). Given the lack of a secondary functional domain to increase the certainty of SpCyp3's orthology to the other members of this group, the different position of SpCyp3 within the dendrogram may potentially indicate that it is not a true member of this orthology group, with it fulfilling a different function that has led to its reduced sequence conservation with the other members of its orthology group.

Another group that appear to have evolved without a distinct common ancestor are the heat shock protein 90 (Hsp90)-associated cyclophilins. hCyp40 is found on a branch amongst the *C. elegans* cyclophilin A-related proteins (Figure 1A). hCyp40 does possess a divergent loop located to one side of the active site which is present in both its cyclophilin 40 orthologues (data not shown) and, more importantly in the context of this observation, in CeCyp3 (Dornan *et al*., 1999). This could in part explain this observation, but the absence of the TPR domain within CeCyp3 still makes the answer to this linkage elusive. Unsurprisingly, both the yeast cyclophilin 40 proteins (SpCyp5 and ScCpr6) are found on the same branch. The *D. melanogaster* cyclophilin 40 (DmCG8336) is found on its own as part of a branch that links it to hCyp40 and which, if traced back, can also be remotely linked to the yeast proteins (Figure 1A). The published research is the only confirmation that SpCyp5, SpCpr6 and hCyp40 are likely to show functional orthology, with DmCD8336 remaining uncharacterized.

Finally, we have the ER-located cyclophilins, which are seen to appear from all three initial branches of the dendrogram (Figure 1A), although all putative cyclophilin B and C orthologues are found diverging from the upper branch, with the exception of the *S. cerevisiae* cyclophilin B orthologue ScCpr5 (central branch). In the upper branch, SpCyp4 is seen to diverge away from the others first, followed by hCypB before CeCyp5 and DmCG2852 diverge. The latter divergence is different from that of the respective species, implying that sequence variation within the *C. elegans* and *D. melanogaster* cyclophilins may have been influenced for similar reasons that are distinct from that of humans. ScCpr5 appears on a branch with only one other distinct protein, ScCpr2, and as this branch appears distinct from all others, it implies that these two cyclophilins evolved independently from a distinct common ancestor within *S. cerevisiae*. The cyclophilin C group appear to have evolved independently of each other, with both being seen to diverge from their respective cyclophilin B orthologue (Figure 1A, upper branch).

Besides the orthology groups identified above by both BLAST and sequence analysis, there remain individual cyclophilins found within the individual repertoires of the organisms in this comparison. The dendrogram (Figure 1A) can in some cases help to shed light on their potential role within the cell through linkage with other groups of known function. An example of this would be that both

Copyright © 2005 John Wiley & Sons, Ltd. 

CeCyp2 and CeCyp3, shown to have no identifiable orthologues by BLAST analysis (Table 4A), are linked by a distinct common ancestor to their cyclophilin A orthologue, CeCyp7. BLAST analysis did show that they have a high similarity to the cyclophilin As, indicating that these are likely to be additional cyclophilin A-like cyclophilins functioning within the nematode. DmCG7768 is found in the same branch as the hCyp33 group (Figure 1A, upper branch), sharing a distinct common ancestor with them. Although it lacks the requisite domains to be a member of this group, looking similar in size and structure to the cyclophilin As, this may indicate its evolution came about by a gene duplication of the DmCyp33 gene, either prior to it gaining the N-terminal RRM domain or in such a fashion that the RRM was lost. hCyp29 appears closely linked to hCypA, both sharing a distinct common ancestor, but has since evolved to be a larger protein with a putative function within the nucleus. Human RanBP2 is also seen as part of the cyclophilin A and 33 branch (Figure 1A, upper branch), with it appearing evolutionarily linked to the cyclophilin 33s, although its size and multidomain structure have led to its localization to the cytoplasmic periphery of the nuclear pore complex (Wu *et al.*, 1995), putatively as a SUMO1 E3 ligase (Pichler *et al.*, 2002). ScCpr7 is a second TPR-containing cyclophilin distinct to *S. cerevisiae* that is found on the same branch as its cyclophilin 40 orthologue ScCpr6 (Figure 1A), with it also reported to interact with Hsp90 (Marsh *et al.*, 1998; Mayr, 2000; Tesic *et al.*, 2003). It appears from this dendrogram that *S. cerevisiae* evolved a second cyclophilin 40-like cyclophilin after its divergence away from the other organisms within this study.

Four of the individual cyclophilins on the upper branch of the dendrogram (Figure 1A) appear to possess a common non-cyclophilin domain. All are RS-cyclophilins that possess what has been termed a 'moca' domain in a published characterization study on the *D. melanogaster* cyclophilin, DmMoca (Cavarec *et al.*, 2002). CeCyp8 and CeCyp9, along with human NK-Cyp, were also reported in this study to possess this 'moca' domain, but in all cases the proteins show no linkage between species, with only the two *C. elegans* cyclophilins appearing to have a recent, and in their case distinct, common ancestor (Figure 1A). hNK-Cyp functions almost solely on the outer cell membrane of natural killer cells (Alkhatib *et al.*, 1997; Anderson *et al.*, 1993; Giardina *et al.*, 1996) as an important component in the recognition of infected cells (Chambers *et al.*, 1994), a process not found in the other compared organisms. The evolution and conservation of this 'moca' domain is therefore elusive when looked at in the context of this dendrogram (Figure 1A) and the function of the proteins that are known to possess it.

Human NK-Cyp also shows linkage to another individual human cyclophilin, hCyp88. Also an RS protein, hCyp88 lacks the 'moca' domain of hNK-Cyp and has been reported to interact with the C-terminal domain of RNA polymerase II (Bourquin *et al.*, 1997) and Cdc28 (Nestel *et al.*, 1996), where it is believed to function in pre-mRNA splicing after co-localizing with splicing factors into nuclear speckles (Bourquin *et al.*, 1997).

The remaining three ER-located cyclophilins are distinct proteins that have evolved, with the exception of ScCpr2, from the lower of the three initial branches (Figure 1A). Two of these individuals are found within *S. cerevisiae*. ScCpr2 is reported as present in the yeast's secretory pathway (Dolinski *et al.*, 1997a; Koser *et al.*, 1991) and induced by heat stress and tunicamycin (Gothel and Marahiel, 1999). It appears linked to its cyclophilin B orthologue, which could imply that it may have evolved from gene duplication and has since gained an individual role within the secretory pathway. The other *S. cerevisiae* protein, ScCpr4, has been reported to localize to the endoplasmic reticulum (Dolinski *et al.*, 1997a), function within the secretory pathway (Gothel and Marahiel, 1999), possess a putative transmembrane domain and to be induced by heat shock and tunicamycin. The remaining ER cyclophilin is *D. melanogaster*'s NinaA (neither inactivation nor after potential A), which is on a branch of its own (Figure 1A). It is expressed solely in the eye (Schneuwly *et al.*, 1989) and is reported as required for visual transduction (Shieh *et al.*, 1989) as an integral membrane protein functioning within the endoplasmic reticulum, as a chaperone in the secretory pathway (Colley *et al.*, 1991; Stamnes *et al.*, 1991) that is required for the correct secretion of the Rh1 subset of rhodopsins (Baker *et al.*, 1994).

Another individual *S. cerevisiae* cyclophilin, ScCpr8, is found linked with ScCpr4 and also CeCyp17 (Figure 1A), and has been reported to be a membrane-bound protein (Franco *et al.*, 1991).

Copyright © 2005 John Wiley & Sons, Ltd.

CeCyp16 appears linked to the CeCyp14 RRM-possessing cyclophilin group, with it reported to be expressed within the anterior and posterior distal portions of the intestine in all larval and adult stages except for the dauer stage, where it is observed in both cell bodies and processes of the ventral chord motor neurons but, interestingly, it was absent from the intestine at these times (Ma *et al.*, 2002).

The three remaining cyclophilins have a putative mitochondrial localization. hmCypD (also referred to as cyclophilin F) is found in the mitochondrial matrix (Bergsma *et al.*, 1991; Connern and Halestrap, 1992; Inoue *et al.*, 1993), with reported functions in the mitochondrial protein-folding machinery (Rassow *et al.*, 1995) and as part of the mitochondrial permeability transition pore complex (Baines *et al.*, 2005; Basso *et al.*, 2005; Halestrap *et al.*, 2002; He and Lemasters, 2002; Lin and Lechleiter, 2002; Nakagawa *et al.*, 2005; Sullivan *et al.*, 1999; Waldmeier *et al.*, 2002). ScCpr3 has been reported as a mitochondrial cyclophilin required for mitochondrial function under heat stress (Dolinski *et al.*, 1997b) and as a protein-folding chaperone within the mitochondria (Davis *et al.*, 1992; Gothel and Marahiel, 1999; Matouschek *et al.*, 1995). The function of CeCyp1 remains unknown, with no published research on its function at the time of writing. The dendrogram (Figure 1A, upper branch) shows that these mitochondrial cyclophilins share very little in common, with each appearing near its respective cyclophilin A orthologue. This observation is supported by their failure to identify each other during BLAST analysis, in which they identify their respective cyclophilin A orthologues instead. Their function may not, therefore, be conserved within their distinct common location within the cell or, if it is, then this most likely came about through convergent evolution.

### FKBP orthology

Table 4D shows that *D. melanogaster* and *H. sapiens* share the greatest number of orthologues, which is closely followed by their orthology to *C. elegans*. All except *D. melanogaster* share just a sole orthologue with the yeasts, with the fruit fly sharing two. This orthology only accounts for at most a quarter of any given higher eukaryotic repertoire or half of a yeast repertoire. It therefore appears that most FKBPs within the repertoires of these organisms are distinct individuals found solely within that repertoire.

Table 4B shows that the only FKBP group to have members in all the compared organisms are those related to hFKBP12. This group has been implicated in transcriptional regulation (Yang *et al.*, 1995), as a regulated inhibitor of tumour growth factor (TGF)-$\beta$ type I signalling (Bryant *et al.*, 1999), as well as in the regulation of the cell cycle (Chen *et al.*, 1997; Okadome *et al.*, 1996; Yao *et al.*, 2000) and calcium release channels (Bultynck *et al.*, 2001a, 2001b; Cameron *et al.*, 1995; Carmody *et al.*, 2001; Wagenknecht *et al.*, 1997). SpFKBP12 has been reported to be important in the early steps of the sexual development pathway of the fission yeast (Weisman *et al.*, 2001), showing that this group appears to have wide-ranging roles.

Only two other groups have a yeast orthologue (Table 4B), with each of the yeasts sharing a single FKBP solely with *D. melanogaster*. SpFKBP39 and DmFKBP39 (Table 4B) are the members of one group, with DmFKBP39 shown to be expressed throughout development (Theopold *et al.*, 1995), and SpFKBP39 has been reported as nuclear (Himukai *et al.*, 1999). A report has implicated this group of cyclophilins in chromatin remodelling involved in ribosomal DNA silencing through a potential role as a histone chaperone (Kuzuhara and Horikoshi, 2004). The second group contains ScFpr2, which has been identified as resident within the ER (Partaledis and Berlin, 1993), but nothing further is known about this group at the time of writing.

There are only three more identified FKBP orthology groups, two related cytoplasmic groups and the other believed to function within the endoplasmic reticulum, despite the varying predicted localizations of its component members (Table 4B). In the latter group hFKBP13 has been reported as resident within the ER (Jin *et al.*, 1991), which is contrary to the PSORT-predicted mitochondrial localization represented in Table 4B, and upregulated in the presence of an increased number of unfolded proteins in the ER (Bush *et al.*, 1994) where it has an apparent role in vesicular trafficking (Padilla *et al.*, 2003).

One of the cytoplasmic groups is found only in the three higher eukaryotes, with the second only found in *D. melanogaster* and humans

Copyright © 2005 John Wiley & Sons, Ltd.

(Table 4B). Both are TPR-possessing FKBPs, with one group having human FKBP52 as a member as well as DmFKBP59, which is reported to be expressed throughout the life-cycle of the fruit fly in the lymph glands, garland cells and oenocyte cells, leading to a proposed function in the exocytic/endocytic pathways that cycle intensively within these tissues (Zaffran, 2000). hFKBP52 (Peattie *et al.*, 1992) is closely related to a member of the second group, hFKBP51 (Sanchez, 1990; Wiederrecht *et al.*, 1992). It has been shown that both bind competitively to the same site on the Hsp90 complex (Nair *et al.*, 1997; Young *et al.*, 1998) and that shuffling between the two effects the subcellular localization and transport of steroid receptors (Davies *et al.*, 2002, 2005; Riggs *et al.*, 2003). The absence of a *C. elegans* orthologue of the hFKBP51 group could potentially indicate a difference in function of the Hsp90-related FKBPs in the nematode.

The remaining FKBPs in the different organisms all appear to be distinct individuals. There are an additional two cytoplasmic TPR containing FKBPs within the human repertoire (Table 4B), hFKBP36 and hFKBP38. hFKBP38 is capable of inhibiting calcineurin in the absence of FK506 (Shirane and Nakayama, 2003), unlike the other FKBPs, suggesting that it functions as a natural inhibitor of the protease, like the previously identified calcineurin inhibitor CAIN (Lai *et al.*, 1998). Its ability to anchor Bcl-2 and Bcl-x(L) to the mitochondria has implicated it in the regulation of apoptosis (Shirane and Nakayama, 2003) and a role in homologous chromosome pairing in meiosis has also been reported (Crackower *et al.*, 2003). *D. melanogaster* also has an additional TPR-containing nuclear FKBP, DmShutdown (Table 4B), which appears to have an essential function in the regulation of germ cell division (Munn and Steward, 2000). The remaining two distinct nuclear FKBPs are both found in *S. cerevisiae* (Table 4B). ScFpr3 has been identified as nuclear (Benton *et al.*, 1994; Manning-Krieg *et al.*, 1994; Shan *et al.*, 1994), as has ScFpr4 (Davey *et al.*, 2000; Dolinski *et al.*, 1997b), with both ScFpr3 and ScFpr4 having been shown to suppress defects seen in the absence of the E3 ubiquitin ligase TOM1 (Davey *et al.*, 2000). SpFKBP39a and CeFkb8 are the only distinct PSORT-predicted cytoplasmic non-human FKBPs (Table 4B). The former has been shown to be nuclear (Himukai *et al.*, 1999) but as yet neither has had any functions identified for it.

The remaining two distinct cytoplasmic FKBPs are all within the human repertoire (Table 4B). hFKBP12.6, which shows 85% similarity to hFKBP12 (Sewell *et al.*, 1994), shares its ability to bind to calcium release channels (Lam *et al.*, 1995; Timerman *et al.*, 1996) and has a proposed role in controlling calcium channel gating through an interaction with cyclic-ADP ribose (Noguchi *et al.*, 1997). hFKBP25 has an N-terminal amphipathic DNA binding helix–loop–helix structure (Hung and Schreiber, 1992; Riviere *et al.*, 1993) and is found to be predominantly nuclear, contrary to its PSORT-predicted cytoplasmic localization, where it has putative roles in cellular control (Jin and Burakoff, 1993), which is supported by its downregulation following p53 induction (Ahn *et al.*, 1999) and transcriptional regulation (Yang *et al.*, 2001).

In total there are four distinct *C. elegans* FKBPs and five human FKBPs that are predicted by PSORT to be endoplasmic reticular (Table 4B). Three of the *C. elegans* FKBPs (dao1 = CeFkb3; dao8 = CeFkb4; dao9 = CeFkb7) have been identified in a study on the DAF-2 insulin receptor-like pathway, which is involved in dauer larva formation, as proteins whose expression is controlled by this pathway (Yu and Larsen, 2001), but quite what their function is remains unknown. The final *C. elegans* FKBP is CeFkb7, whose function remains unknown, although the presence of a calcium-binding EF-hand motif in its C-terminal region may indicate that its function is regulated by intracellular calcium levels (Honore and Vorum, 2000).

Besides hFKBP65, which has been reported as localized within the ER lumen of cells only during the growth and development of tissues when it appears to function as a protein chaperone (Patterson *et al.*, 2000), the remaining distinct human FKBPs have yet to have functions assigned to them. The presence of a calcium-binding EF-hand motif in its C-terminal region of both hFKBP60 and hFKBP65 may indicate that their function, like that of CeFkb7, is regulated by intracellular calcium levels (Honore and Vorum, 2000).

The dendrogram generated from the FKBP sequences (Figure 1B) only tentatively supports the BLAST-identified orthology groups between the FKBP repertoires. The group containing

Copyright © 2005 John Wiley & Sons, Ltd. 

SpFKBP39 and DmFKBP39 does appear in the same branch of the dendrogram, which also contains SpFKBP39a, confirming that the two 39 kDa *Sz. pombe* FKBPs are related. This branch also contains the two distinct nuclear *S. cerevisiae* FKBPs, ScFpr3 and ScFpr4, and hFKBP25, which has been reported to be nuclear (Jin and Burakoff, 1993), contrary to its PSORT-predicted localization. All but one of the nuclear FKBPs therefore appears to have evolved from a single common ancestor, with the exception being DmShutdown. The TPR-containing group, which has hFKBP52 as a member, all appear on the same branch of the dendrogram, with a distinct common ancestor shared with hFKBP51, which appears to diverge from hFKBP52 after their common ancestor diverged from the other members of the hFKBP52 group. The other member of the hFKBP51 group appears on a separate branch closely associated with the TPR-containing hFKBP38 and more loosely with hFKBP36 and DmShutdown. The only endoplasmic reticular group containing ScFpr2 and DmCG14715 appears on the same branch diverging from a distinct common ancestor, with hFKBP22, hFKBP23 and DmFKBP13 also found linked on this branch. DmFKBP13 is separated from the rest of its orthology group, which are located on another branch along with all but one of the distinct *C. elegans* endoplasmic reticular FKBPs, the exception being CeFkb7, which appears on a separate branch on its own. Finally we have the FKBP12 group, which are found in two different locations. ScFpr1 and SpFKBP12 are found on the same branch, diverging from a distinct common ancestor, and linked to their other FKBPs, with the exception of ScFpr2. The remaining FKBP12s all diverge from a single branch in the same order as their species diverged, but they share their common ancestor with the hFKBP52 group.

Despite the dendrogram's lack of support for the orthology groups, it does imply that there is more significance in their location within the cell and the domains they possess than in orthologous function. All the cytoplasmic and nuclear FKBPs appear to have evolved from the upper branch of the dendrogram (Figure 1B), with all the endoplasmic reticular FKBPs, with the exception of hFKBP19, appearing to evolve from the lower branch of the dendrogram, although CeFkb7 appears to have evolved independently. Within each of the major branches, the subbranches can be seen to group such that those FKBPs with the same domains present have largely evolved from the same branch or group of linked branches.

### Parvulin orthology

Compared with the cyclophilin and FKBP repertoires, the parvulin repertoires are relatively small. All the compared organisms share a single parvulin in common, with a second parvulin only shared between the higher eukaryotes (Table 4D). The sole parvulin they all share in common is that of the hPin1 group identified by BLAST analysis (Table 4C).

hPin1 has been reported to specifically isomerize only phosphorylated serine/threonine–proline bonds (Lu *et al.*, 2002), making it likely that all its functions can be linked to a regulatory role with phosphoproteins. Its catalytic activity has been implicated in the restoration of the function of the phosphorylated-neuronal Tau protein (Lu *et al.*, 1999; Zhou *et al.*, 2000) and in maintaining Bcl2 in a phosphorylated state (Basu *et al.*, 2002). It has been reported to have a critical regulatory role in the function of p53 (Wulf *et al.*, 2002; Zacchi *et al.*, 2002; Zheng *et al.*, 2002), a regulatory role in transcription (Albert *et al.*, 1999; Wen and Shatkin, 1999), a role in the cell cycle $G_2$–M progression (Crenshaw *et al.*, 1998) and with a potential function also reported in mitosis (Messenger *et al.*, 2002). Interestingly, the cytoplasmic component of the transcription factor NF-AT (nuclear factor of activated T-cells), when in a phosphorylated form, interacts with Pin1's WW domain and this interaction stops NF-ATc being dephosphorylated by the $Ca^{2+}$-dependent protein phosphatase calcineurin (Liu *et al.*, 2001), indicating a role in the regulation of NF-AT signalling. The immunosuppressive drugs cyclosporin A and FK506 both cause immunosuppression by this mechanism (Schreiber and Crabtree, 1992), making it appear that Pin1 has a role in the regulation of the immune response.

DmDodo has also been reported as involved in signal transduction (Maleszka *et al.*, 1997), protein folding (Maleszka *et al.*, 1997) and, more recently, as a MAP kinase signal responder during oogenesis (Hsu *et al.*, 2001).

Of the yeast Pin1 orthologues, SpPin1 is believed to be it a positive regulator of the cell cycle control proteins Wee1 and Cdc25 (Huang *et al.*,

Copyright © 2005 John Wiley & Sons, Ltd. 

2001). ScEss1 is reported as nuclear and is involved in transcription (Kops *et al.*, 2002; Morris *et al.*, 1999; Wilcox *et al.*, 2004; Wu *et al.*, 2000, 2003; Xu *et al.*, 2003), cell cycle regulation (Huang *et al.*, 2001) and is essential for vegetative growth (Hanes *et al.*, 1989). Interestingly, ScEss1 has been reported as an essential gene (Hanes *et al.*, 1989), whereas SpPin1 (Huang *et al.*, 2001), DmDodo (Maleszka *et al.*, 1996) and *Cryptococcus neoformans* Ess1 (Ren *et al.*, 2005) have been shown to be non-essential. Cross-talk between ScEss1 and ScCpr1 (Fujimori *et al.*, 2001), its hCypA orthologue, has been shown to modulate the activity of the Sin3–Rpd3 complex, with excess histone deacetylation causing mitotic arrest in ScEss1 mutants (Arevalo-Rodriguez *et al.*, 2000), and CnEss1-null mutants have been reported to be hypersensitive to cyclosporin A (CsA; Ren *et al.*, 2005), suggesting a cyclophilin-mediated redundancy mechanism. Disruption of ScEss1 can be complemented by DmDodo (Maleszka *et al.*, 1996) and the plant *Digitalis lanata*'s Par13 (Metzner *et al.*, 2001), which lacks the WW domain conserved in the other proteins, indicating that a conserved functionality may exist between all Pin1 orthologues that is essential in some but not all organisms under normal growth conditions.

The parvulin orthology group found only within the higher eukaryotes are related to hPar14 (EPVH; Table 4C), which has been reported in two different studies to localize within two different areas of the cell, preferentially within the mitochondrial matrix (Rulten *et al.*, 1999) and preferentially within the nucleus (Uchida *et al.*, 1999). It has been reported to be part of the preribosomal ribonucleoprotein (pre-rRNP) complexes and as interacting with fibronectin, p160 (Myb-binding), p58 cyclin-dependant kinase (a $G_2$/M-specific protein kinase) and $\alpha$- and $\beta$-tubulin (Fujiyama *et al.*, 2002). The PSORT-predicted cytoplasmic localization of both hPar14 and DmCG11858 is in contrast to the predicted nuclear localization of CePin2. Given the apparent functions of this group, a nuclear localization is more likely, contrary to the reported mitochondrial localization, indicating that the localization events may be due more to interactions with other molecules than sequence motifs.

The only distinct parvulin is in the repertoire of *D. melanogaster*, DmCG32845. No orthologues were identified by BLAST searching of the currently available sequence databases, indicating that it may be unique to the fruit fly. It is just over twice the size of the hPin1 group but only a single parvulin-like rotamase domain is identified within its sequence (data not shown). It has a predicted nuclear localization but it has not been previously reported, making this is a novel identification in this study. Research into its function will be invaluable to see what function this parvulin performs solely within *D. melanogaster*, thus indicating reasons for its absence in the other organisms. The dendrogram for the parvulins (Figure 1C) implies that this novel parvulin is more closely related to the hPar14 group, which also shares its branch with the *D. melanogaster* hPin1 orthologue, DmDodo. It therefore appears that the evolution of the parvulins of *D. melanogaster* is more closely linked than those of the other compared organisms. hPin1 appears to have evolved in a more independent fashion, with the remaining hPin1 orthologues appearing to evolve from a distinct common ancestor, with SpPin1 and ScEss1 unsurprisingly sharing a distinct common ancestor themselves.

### Discussion

Of the identified PPIase repertoires, humans unsurprisingly possess the greatest number, although fewer than we would expect, given the trend of PPIase numbers vs. genes seen with the organisms in the Table 3. A repertoire size of ca. 40 would be more in keeping with its genome size. Looking at the component PPIase member numbers that make up their repertoires, humans possess a lower number of cyclophilins than we would expect but its number of FKBPs and parvulins are as expected, indicating that its smaller than expected PPIase repertoire can be accounted for by a smaller cyclophilin family. With two parvulins found in the human and *C. elegans* repertoires and only a single parvulin found in both of the yeasts, *D. melanogaster* is the only known eukaryote to have three parvulins, with its third parvulin appearing to be unique.

We have shown that the repertoires of these organisms have both members with common function and those which appear distinct for any given organism. Interestingly, the cyclophilin repertoire of *Sz. pombe* has no unique members and the two yeasts share no PPIases that are unique to themselves. The proportion of cyclophilins and

Copyright © 2005 John Wiley & Sons, Ltd. 

parvulins with identified orthologues within the repertoires is high, whereas the proportion seen with the FKBPs is low by comparison. It therefore appears that the cyclophilins and parvulins have evolved to perform conserved functions, while the FKBPs have evolved to fill ever-changing niches within these constantly evolving organisms.

There were a total of 12 distinct cyclophilin orthology groupings identified by BLAST analysis, and confirmed in most cases by sequence analysis, with eight identified domain architectures between them. Only four of theses groups have an *S. cerevisiae* member, whereas nine have members in its fellow yeast *Sz. pombe*, leaving only three that are unique to the repertoires of the multicellular organisms. *S. cerevisiae* lacks members of three cytoplasmic groups, which include those involved in transcriptional regulation, pre-mRNA splicing and signal transduction, and it also lacks members of two nuclear groups believed to be involved in transcriptional regulation as well as cell morphogenesis, cortical organization and nuclear reorganization. Those orthology groups that lack a *Sz. pombe* and *S. cerevisiae* member appear to function in pathways that would not be found in single-celled yeasts, such as the control of cell differentiation, or show differential expression linked to cell type.

Although no unique cyclophilins are present in the *Sz. pombe* repertoire, there are five in that of its fellow yeast *S. cerevisiae*, which equals the number in humans and is only two less than are found in *C. elegans*. *D. melanogaster* has only three, one in each of the cytoplasmic, nuclear and endoplasmic reticular compartments, with two of these appearing to be involved in pathways specific to the fruit fly and one being similar to its cytoplasmic cyclophilin A orthologue. One of the *S. cerevisiae* unique cyclophilins appears to be a second member of the TPR-possessing cyclophilin 40 group only found within this yeast, with another two unique cyclophilins found within its endoplasmic reticulum. The final two unique *S. cerevisiae* cyclophilins have distinct localizations, one to the plasma membrane and the other within the mitochondria. The latter has compatriots in humans and *C. elegans*, although these appear unrelated. No mitochondrial cyclophilin is found in either *Sz. pombe* or *D. melanogaster*. Besides this mitochondrial cyclophilin, *C. elegans* also has three unique cyclophilins in both the nucleus and cytoplasm. Two of the latter appear to be additional cyclophilin A-like cyclophilins, with the third appearing to be involved in cell morphogenesis and cortical organization. The former group has one of unknown function, with the remaining two appearing to be part of a family that includes a unique protein in both *D. melanogaster* and human. These appear to all possess what has been termed a 'moca' domain, but their function remains largely unknown. All of the five extra human cyclophilins, which includes the 'moca'-possessing protein, are located in the nucleus with the exception of the mitochondrial cyclophilin mentioned above. The final cyclophilin is hRanBP2, a large multidomain cyclophilin that functions as part of the nuclear pore complex.

*S. cerevisiae* appears to require a greater number within the endoplasmic reticulum than any of the other organisms compared, implying that either their function may have been incorporated into the PPIases they possess within that cellular structure, or that their function is not required in these other organisms. The lack of a mitochondrial cyclophilin in *Sz. pombe* is surprising, given its presence in all the others with the exception of *D. melanogaster*. It therefore appears that mitochondria in *Sz. pombe* share a greater similarity with those in *D. melanogaster* in this respect. However, a single *Neurospora crassa* cyclophilin gene has been reported to encode both a cytosolic and a mitochondrial isoform (Tropschug *et al.*, 1988), which could possibly explain the absence of a dedicated mitochondrial cyclophilin within *Sz. pombe* and *D. melanogaster*. The isolation of a cyclophilin from within the mitochondria of both these organisms would therefore clarify this.

The FKBP repertoires of the compared organisms show less orthology than was seen with the cyclophilin repertoires, with the yeasts showing approximately 50% orthology with the higher eukaryotes, which themselves show little more than a 20% orthology with each other. A majority of FKBPs therefore appear to be distinct to any given organism, implying that their function is organism-specific in most cases, with the sole FKBP they all share in common being the FKBP12 family. This family appears to have a wide range of functions within cell cycle regulation, calcium release and transcriptional regulation that appear to place the function of the FKBP12 group predominantly within intracellular signalling. Whilst the functions of the additional FKBPs in the two yeasts are

Copyright © 2005 John Wiley & Sons, Ltd. 

largely unknown, those in the multicellular eukaryotes appear to function either in processes, such as the growth and differentiated development of tissues, or pathways, such as the anchoring of Bcl-proteins to the mitochondria, that are probably not required/present within the yeasts.

Unlike with the cyclophilins, sequence analysis did not support many of the BLAST-identified FKBP orthology groups. Although most groups clustered into the same regions within the dendrogram, many were not able to be traced back to a distinct common ancestor. The main organization of the dendrogram appeared to be based upon their secondary domains and localization rather than by orthologous function.

In contrast to the cyclophilin and FKBP repertoires, the number of parvulins within the compared organisms is small. They all share a single parvulin in common, orthologues of hPin1, with the higher eukaryotes sharing an additional parvulin in common related to hPar14. The hPin1 orthology group appear to function in a wide range of processes from intracellular signalling to the regulation of transcription and the cell cycle, with some appearing essential for cell survival, whilst the hPar14 group also appear to potentially function in some intracellular signalling pathways as well as within the ribosomal processes. The reason for the absence of a hPar14 orthologue within the genomes of both the yeasts cannot easily be explained by their function, making the most likely explanation that, while the yeasts can cope with just a single parvulin, the evolution of the higher eukaryotes has required an additional parvulin to either share the work-load or to fill a particular niche that requires a parvulin of divergent form to that of hPin1. A novel parvulin that identified no orthologues in the presently available sequence databases is present solely within the repertoire of *D. melanogaster* and appears, based on sequence analysis, to be a distant relative of the hPar14 group, whilst evolving to an unknown function that currently appears to be required solely within the fruit fly. Analysis of the function of this parvulin will therefore be of great interest in unlocking why it is present solely within the fruit fly.

Looking at the global localization patterns of the three PPIase families within the compared organisms (Table 4A–C), the cyclophilins appear predominantly within the cytoplasm and nucleus, with only a few present within the endoplasmic reticulum. *Sz. pombe* has just a single cyclophilin within the endoplasmic reticulum, unlike the other compared organisms that have two, with the exception of *S. cerevisiae* which has three. They all share a single ER-resident cyclophilin in common, while the remaining ER cyclophilins in both *S. cerevisiae* and *D. melanogaster* are specific to each of them, with only the additional cyclophilin in human and *C. elegans* appearing orthologous.

The FKBPs, however, reside predominantly within the endoplasmic reticulum and cytoplasm. Nuclear FKBPs are only present within the repertoires of the two yeasts and *D. melanogaster*, making these distinct from those of humans and *C. elegans*, which possess a greater number within the endoplasmic reticulum, although these appear specific to each of them. *Sz. pombe* is unique in lacking an FKBP within the endoplasmic reticulum, unlike *S. cerevisiae*, which has a single one present. This greater localization to the vesicular pathway could explain the lack of orthology observed within the FKBP family of the compared organisms, which we have attributed to their potential evolution to serve in variable niches within the different organisms. As the number of genes in any given organism increases, so do the number of proteins, and with that an increase in the number of proteins requiring chaperoning is to be expected. Given the location of the FKBPs within the protein-folding pathway and the apparent linkage between the number present in any given organism and its number of genes (Table 3), it could be that they are evolving to serve this increased requirement for chaperoning, driven independently within each organism, thus leading to their observed lack of orthology. Taking this into consideration, we would hypothesize that the cyclophilins are evolving to perform specific conserved functions within the different organisms, while the FKBPs are evolving, in most cases, to meet the more individual needs for protein chaperoning. The presence of nuclear FKBPs within the two yeasts and *D. melanogaster* could be examples of functions initially performed by the FKBPs that have since evolved to be filled by cyclophilins within *C. elegans* and humans. The lack of an *Sz. pombe* FKBP within the endoplasmic reticulum and the lack of any compensatory cyclophilins does, however, indicate that that the true reasons behind this may be more complicated

Copyright © 2005 John Wiley & Sons, Ltd.

than the simplified hypothesis we have proposed here.

The parvulins appear to be a family of PPIases found solely within the nucleus. They all share a single parvulin in common, with the higher eukaryotes also sharing a second smaller parvulin. Their greater presence in the prokaryotes, where in some cases they are the largest or sole PPIase family present (data not shown), makes it appear that their evolution has been such that the cyclophilins and FKBPs have replaced them in their function or that their functions have evolved such that they do not require them.

This comparison has shown that, while the PPIase repertoire of *S. cerevisiae* has been the subject of a great deal of research to identify their functions within the cell, it is a poor representative of the repertoires of the more complex organisms. In contrast, its fellow yeast *Sz. pombe* appears to be a good model organism for the study of two of the three PPIase families, the cyclophilins and parvulins, with it not appearing to be a good system for the study of the FKBP family. This lack of orthology appears global in the FKBP repertoires, implying that they function in a capacity that is specific to each organism. Thus, *Sz. pombe* represents an excellent single-celled model organism for the study of the functions of the different PPIase families, which can be related to the function of their orthologues within more complex eukaryotes.

### Acknowledgements

Trevor Pemberton was in receipt of a BBSRC Studentship at the time of this research. The authors would like to thank Professor Andrew Smith (University of Sussex) and Dr. Peter Klappa (University of Kent) for their help in the preparation of this manuscript.

### References

Adams MD. 2000. The genome sequence of *Drosophila melanogaster*. *Science* **287**: 2185–2195.

Ahn J, Murphy M, Kratowicz S *et al*. 1999. Downregulation of the stathmin/Op18 and FKBP25 genes following p53 induction. *Oncogene* **18**: 5954–5958.

Albert A, Lavoie S, Vincent M. 1999. A hyperphosphorylated form of RNA polymerase II is the major interphase antigen of the phosphoprotein antibody MPM-2 and interacts with the peptidyl-prolyl isomerase Pin1. *J Cell Sci* **112**: 2493–2500.

Alkhatib G, Murata K, Roder JC. 1997. Cellular distribution of a natural killer cell tumour recognition-related surface antigen in purified human lymphocytes. *Immunology* **92**: 173–179.

Allain F, Denys A, Spik G. 1994. Characterisation of surface binding sites for cyclophilin B on a human tumour T-Cell line. *J Biol Chem* **269**: 16 537–16 546.

Altschul SF, Madden TL, Schaffer AA *et al*. 1997. Gapped BLAST and PSI-BLAST: a new generation of protein database search programs. *Nucleic Acids Res* **25**: 3389–3402.

Anderson M, Fair K, Amero S *et al*. 2002. A new family of cyclophilins with an RNA recognition motif that interact with members of the trx/MLL protein family in *Drosophila* and human cells. *Dev Genes Evol* **212**: 107–113.

Anderson SK, Gallinger S, Roder J *et al*. 1993. A cyclophilin-related protein involved in the function of natural killer cells. *Proc Natl Acad Sci USA* **90**: 542–546.

Ansari H, Greco G, Luban J. 2002. Cyclophilin A peptidyl-prolyl isomerase activity promotes Zpr1 nuclear export. *Mol Cell Biol* **22**: 6993–7003.

Arevalo-Rodriguez M, Cardenas ME, Wu X, Hanes SD, Heitman J. 2000. Cyclophilin A and Ess1 interact with and regulate silencing by the Sin3-Rpd3 histone deacetylase. *EMBO J* **19**: 3739–3749.

Arevalo-Rodriguez M, Heitman J. 2005. Cyclophilin A is localized to the nucleus and controls meiosis in *Saccharomyces cerevisiae*. *Eukaryot Cell* **4**: 17–29.

Baines CP, Kaiser RA, Purcell NH *et al*. 2005. Loss of cyclophilin D reveals a critical role for mitochondrial permeability transition in cell death. *Nature* **434**: 658.

Baker EK, Colley NJ, Zuker CS. 1994. The cyclophilin homolog NinaA functions as a chaperone, forming a stable complex *in vivo* with its protein target rhodopsin. *EMBO J* **13**: 4886–4895.

Basso E, Fante L, Fowlkes J *et al*. 2005. Properties of the permeability transition pore in mitochondria devoid of cyclophilin D. *J Biol Chem* **280**: 18 558–18 561.

Basu A, Das M, Qanungo S *et al*. 2002. Proteasomal degradation of human peptidyl prolyl isomerase pin1-pointing phospho Bcl2 toward dephosphorylation. *Neoplasia* **4**: 218–227.

Benton BM, Zang JH, Thorner J. 1994. A novel FK506- and rapamycin-binding protein (FPR3 gene product) in the yeast *Saccharomyces cerevisiae* is a proline rotamase localized to the nucleolus. *J Cell Biol* **127**: 623–639.

Bergsma DJ, Eder C, Gross M *et al*. 1991. The cyclophilin multigene family of peptidyl-prolyl isomerases. Characterization of three separate human isoforms. *J Biol Chem* **266**: 23 204–23 214.

Bharadwaj S, Ali A, Ovsenek N. 1999. Multiple components of the HSP90 chaperone complex function in regulation of heat shock factor 1 *in vivo*. *Mol Cell Biol* **19**: 8033–8041.

Birnby DA, Link EM, Vowels JJ *et al*. 2000. A transmembrane guanylyl cyclase (DAF-11) and Hsp90 (DAF-21) regulate a common set of chemosensory behaviors in *Caenorhabditis elegans*. *Genetics* **155**: 85–104.

Birney E, Kumar S, Krainer AR. 1993. Analysis of the RNA-recognition motif and RS and RGG domains: conservation in metazoan pre-mRNA splicing factors. *Nucleic Acids Res* **21**: 5803–5816.

Bose S, Mucke M, Freedman RB. 1994. The characterization of a cyclophilin-type peptidyl prolyl *cis-trans*-isomerase from the endoplasmic-reticulum lumen. *Biochem J* **300**: 871–875.

Bourquin J, Stagljar I, Meier P *et al*. 1997. A serine/arginine-rich nuclear matrix cyclophilin interacts with the C-terminal domain of RNA polymerase II. *Nucleic Acids Res* **25**: 2055–2061.

Copyright © 2005 John Wiley & Sons, Ltd. 

Brazin KN, Mallis RJ, Fulton DB, Andreotti AH. 2002. Regulation of the tyrosine kinase Itk by the peptidyl-prolyl isomerase cyclophilin A. *Proc Natl Acad Sci USA* **99**: 1899–1904.

Brown CR, Cui D-Y, Hung GG-C, Chiang H-L. 2001. Cyclophilin A mediates Vid22p function in the import of fructose-1,6-bisphosphatase into Vid vesicles. *J Biol Chem* **276**: 48 017–48 026.

Bryant Z, Subrahmanyan L, Tworoger M *et al.* 1999. Characterization of differentially expressed genes in purified *Drosophila* follicle cells: toward a general strategy for cell type-specific developmental analysis. *Proc Natl Acad Sci USA* **96**: 5559–5564.

Bukrinsky MI. 2002. Cyclophilins: unexpected messengers in intracellular communications. *Trends Immunol* **23**: 323–325.

Bultynck G, De Smet P, Rossi D *et al.* 2001a. Characterisation and mapping of the 12 kDa FK506-binding protein (FKBP12)-binding site on different isoforms of the ryanodine receptor and of the inositol 1,4,5-trisphosphate receptor. *Biochem J* **354**: 413–422.

Bultynck G, Rossi D, Callewaert G *et al.* 2001b. The conserved sites for the FK506-binding proteins in ryanodine receptors and inositol 1,4,5-trisphosphate receptors are structurally and functionally different. *J Biol Chem* **276**: 47 715–47 724.

Bush KT, Hendrickson BA, Nigam SK. 1994. Induction of the FK506-binding protein, FKBP13, under conditions which misfold proteins in the endoplasmic reticulum. *J Biochem* **303**: 705–708.

Cameron A, Steiner J, Sabatini D *et al.* 1995. Immunophilin FK506 binding protein associated with inositol 1,4,5-trisphosphate receptor modulates calcium flux. *Proc Natl Acad Sci USA* **92**: 1784–1788.

Cande C, Vahsen N, Kouranti I *et al.* 2004. AIF and cyclophilin A cooperate in apoptosis-associated chromatinolysis. *Oncogene* **23**: 1514–1521.

Carmody M, Mackrill JJ, Sorrentino V, O'Neill C. 2001. FKBP12 associates tightly with the skeletal muscle type 1 ryanodine receptor, but not with other intracellular calcium release channels. *FEBS Lett* **505**: 97–102.

Cavarec L, Kamphausen T, Dubourg B *et al.* 2002. Identification and characterization of Moca-cyp: a *Drosophila melanogaster* nuclear cyclophilin. *J Biol Chem* **277**: 41 171–41 182.

Chambers CA, Gallinger S, Anderson SK *et al.* 1994. Expression of the NK-TR gene is required for NK-like activity in human T cells. *J Immunol* **152**: 2669–2674.

Chen Y-G, Liu F, Massague J. 1997. Mechanism of TGF-ß receptor inhibition by FKBP12. *EMBO J* **16**: 3866–3876.

Colley NJ, Baker EK, Stamnes MA, Zuker CS. 1991. The cyclophilin homolog ninaA is required in the secretory pathway. *Cell* **67**: 255–263.

Connern CP, Halestrap AP. 1992. Purification and N-terminal sequencing of peptidyl-prolyl *cis-trans*-isomerase from rat liver mitochondrial matrix reveals the existence of a distinct mitochondrial cyclophilin. *J Biochem* **284**: 381–385.

The C. elegans Genome Consortium. 1998. Genome sequence of the nematode *C. elegans*: a platform for investigating biology. *Science* **282**: 2012–2018.

Crackower MA, Kolas NK, Noguchi J *et al.* 2003. Essential role of Fkbp6 in male fertility and homologous chromosome pairing in meiosis. *Science* **300**: 1291–1295.

Crenshaw DG, Yang J, Means AR, Kornbluth S. 1998. The mitotic peptidyl-prolyl isomerase, Pin1, interacts with Cdc25 and Plx1. *EMBO J* **17**: 1315–1327.

Davey M, Hannam C, Wong C, Brandl CJ. 2000. The yeast peptidyl proline isomerases FPR3 and FPR4, in high copy numbers, suppress defects resulting from the absence of the E3 ubiquitin ligase TOM1. *Mol Gen Genet* **263**: 520–526.

Davies TH, Ning Y-M, Sanchez ER. 2002. A new first step in activation of steroid receptors: hormone-induced switching of FKBP51 and FKBP52 immunophilins. *J Biol Chem* **277**: 4597–4600.

Davies TH, Ning YM, Sanchez ER. 2005. Differential control of glucocorticoid receptor hormone-binding function by tetratricopeptide repeat (TPR) proteins and the immunosuppressive ligand FK506. *Biochemistry* **44**: 2030–2038.

Davis ES, Becker A, Heitman J, Hall MN, Brennan MB. 1992. A yeast cyclophilin gene essential for lactate metabolism at high temperature. *Proc Natl Acad Sci USA* **89**: 11 169–11 173.

Davis J, Boswell B, Bachinger H. 1989. Thermal stability and folding of type IV procollagen and effect of peptidyl-prolyl *cis-trans*-isomerase on the folding of the triple helix. *J Biol Chem* **264**: 8956–8962.

Devasahayam G, Chaturvedi V, Hanes SD. 2002. The Ess1 prolyl isomerase is required for growth and morphogenetic switching in *Candida albicans*. *Genetics* **160**: 37–48.

Dolinski K, Muir S, Cardenas M, Heitman J. 1997a. All cyclophilins and FK506 binding proteins are, individually and collectively, dispensable for viability in *Saccharomyces cerevisiae*. *Proc Natl Acad Sci USA* **94**: 13 093–13 098.

Dolinski K, Scholz C, Muir RS *et al.* 1997b. Functions of FKBP12 and mitochondrial cyclophilin active site residues *in vitro* and *in vivo* in *Saccharomyces cerevisiae*. *Mol Biol Cell* **8**: 2267–2280.

Dornan J, Page AP, Taylor P *et al.* 1999. Biochemical and structural characterization of a divergent loop cyclophilin from *Caenorhabditis elegans*. *J Biol Chem* **274**: 34 877–34 883.

Duina AA, Kalton HM, Gaber RF. 1998. Requirement for Hsp90 and a CyP-40-type cyclophilin in negative regulation of the heat shock response. *J Biol Chem* **273**: 18 974–18 978.

Edgar KA, Belvin M, Parks AL *et al.* 2005. Synthetic lethality of retinoblastoma mutant cells in the *Drosophila* eye by mutation of a novel peptidyl prolyl isomerase gene. *Genetics* Genetics **170**: 161–171.

Eisenmesser EZ, Bosco DA, Akke M, Kern D. 2002. Enzyme dynamics during catalysis. *Science* **295**: 1520–1523.

Franco L, Jimenez A, Demolder J *et al.* 1991. The nucleotide sequence of a third cyclophilin-homologous gene from *Saccharomyces cerevisiae*. *Yeast* **7**: 971–979.

Friedman J, Trahey M, Weissman I. 1993. Cloning and characterization of cyclophilin C-associated protein: a candidate natural cellular ligand for cyclophilin C. *Proc Natl Acad Sci USA* **90**: 6815–6819.

Friedman J, Weissman I, Friedman J, Alpert S. 1994. An analysis of the expression of cyclophilin C reveals tissue restriction and an intriguing pattern in the mouse kidney. *Am J Pathol* **144**: 1247–1256.

Frigerio G, Pelham HRB. 1993. A *Saccharomyces cerevisiae* cyclophilin resident in the endoplasmic reticulum. *J Mol Biol* **233**: 183–188.

Copyright © 2005 John Wiley & Sons, Ltd. 

Fujimori F, Gunji W, Kikuchi J *et al*. 2001. Crosstalk of prolyl isomerases, Pin1/ESS1, and cyclophilin A. *Biochem Biophys Res Commun* **289**: 181–190.

Fujiyama S, Yanagida M, Hayano T *et al*. 2002. Isolation and proteomic characterization of human parvulin-associating preribosomal ribonucleoprotein complexes. *J Biol Chem* **277**: 23 773–23 780.

Galat A. 1993. Peptidyl proline *cis-trans*-isomerases: immunophilins. *Eur J Biochem* **216**: 689–707.

Galat A. 1999. Variations of sequences and amino acid compositions of proteins that sustain their biological functions: an analysis of the cyclophilin family of proteins. *Arch Biochem Biophys* **371**: 149–162.

Galat A. 2004. Function-dependent clustering of orthologues and paralogues of cyclophilins. *Proteins* **56**: 808–820.

Galat A, Metcalfe SM. 1995. Peptidylproline *cis/trans* isomerases. *Progr Biophys Mol Biol* **63**: 67–118.

Geer LY, Domrachev M, Lipman DJ, Bryant SH. 2002. CDART: protein homology by domain architecture. *Genome Res* **12**: 1619–1623.

Giardina SL, Coffman JD, Young HA *et al*. 1996. Association of the expression of an SR-cyclophilin with myeloid cell differentiation. *Blood* **87**: 2269–2274.

Gish W, Altschul S, Webb M, Myers E, Lipman D. 1990. Basic local alignment search tool. *J Mol Biol* **215**: 403–410.

Goes FS, Martin J. 2001. Hsp90 chaperone complexes are required for the activity and stability of yeast protein kinases Mik1, Wee1 and Swe1. *Eur J Biochem* **268**: 2281–2289.

Goffeau A, Barrell BG, Bussey H *et al*. 1996. Life with 6000 genes. *Science* **274**: 563–567.

Gothel SF, Marahiel MA. 1999. Peptidyl-prolyl *cis-trans* isomerases, a superfamily of ubiquitous folding catalysts. *Cell Mol Life Sci* **55**: 423–436.

Gothel SF, Scholz C, Schmid FX, Marahiel MA. 1998. Cyclophilin and trigger factor from *Bacillus subtilis* catalyze *in vitro* protein folding and are necessary for viability under starvation conditions. *Biochemistry* **37**: 13 392–13 399.

Halestrap AP, Davidson AM. 1990. Inhibition of $Ca^{2+}$-induced large-amplitude swelling of liver and heart mitochondria by cyclosporin is probably caused by the inhibitor binding to mitochondrial-matrix peptidyl-prolyl *cis-trans* isomerase and preventing it interacting with the adenine nucleotide translocase. *Biochem J* **268**: 153–160.

Halestrap AP, McStay GP, Clarke SJ. 2002. The permeability transition pore complex: another view. *Biochimie* **84**: 153–166.

Handschumacher RE, Harding MW, Rice J, Drugge RJ, Speicher DW. 1984. Cyclophilin: a specific cytosolic binding protein for cyclosporin A. *Science* **226**: 544–547.

Hanes SD, Shank PR, Bostian KA. 1989. Sequence and mutational analysis of ESS1, a gene essential for growth in *Saccharomyces cerevisiae*. *Yeast* **5**: 55–72.

Harding M, Handschumacher R, Speicher D. 1986. Isolation and amino acid sequence of cyclophilin. *J Biol Chem* **261**: 8547–8555.

Harrison R, Stein RL. 1992. Mechanistic studies of enzymatic and nonenzymatic prolyl *cis-trans* isomerization. *J Am Chem Soc* **114**: 3464–3471.

He L, Lemasters JJ. 2002. Regulated and unregulated mitochondrial permeability transition pores: a new paradigm of pore structure and function? *FEBS Lett* **512**: 1–7.

He Z, Li L, Luan S. 2004. Immunophilins and parvulins. Superfamily of peptidyl prolyl isomerases in *Arabidopsis*. *Plant Physiol* **134**: 1248–1267.

Hemenway C, Heitman J. 1993. Proline isomerases in microorganisms and small eukaryotes. *Ann NY Acad Sci* **696**: 38–46.

Himukai R, Kuzuhara T, Horikoshi M. 1999. Relationship between the subcellular localization and structures of catalytic domains of FKBP-type PPIases. *J Biochem Tokyo* **126**: 879–888.

Honore B, Vorum H. 2000. The CREC family, a novel family of multiple EF-hand, low-affinity $Ca^{2+}$-binding proteins localized to the secretory pathway of mammalian cells. *FEBS Lett* **466**: 11–18.

Horibe T, Yosho C, Okada S *et al*. 2002. The chaperone activity of protein disulfite isomerase is affected by cyclophilin B and cyclosporin A *in vitro*. *J Biochem Tokyo* **132**: 401–407.

Horowitz DS, Kobayashi R, Krainer AR. 1997. A new cyclophilin and the human homologues of yeast Prp3 and Prp4 form a complex associated with U4/U6 snRNPs. *RNA* **3**: 1374–1387.

Horowitz DS, Lee EJ, Mabon SA, Misteli T. 2002. A cyclophilin functions in pre-mRNA splicing. *EMBO J* **21**: 470–480.

Horton P, Nakai K. 1996. A probabilistic classification system for predicting the cellular localization sites of proteins. *Proceedings of the 4th International Conference on Intelligent Systems Molecular Biology*, St. Louis, USA **4:** 109–115.

Horton P, Nakai K. 1997. Better prediction of protein cellular localization sites with the k nearest neighbors classifier. *Proceedings of the 5th International Conference on Intelligent Systems for Molecular Biology*, Halkidiki, Greece **5:** 147–152.

Hsu T, McRackan D, Vincent TS, Gert de Couet H. 2001. *Drosophila* Pin1 prolyl isomerase Dodo is a MAP kinase signal responder during oogenesis. *Nat Cell Biol* **3**: 538–543.

Huang HK, Forsburg SL, John UP, O'Connell MJ, Hunter T. 2001. Isolation and characterization of the Pin1/Ess1p homologue in *Schizosaccharomyces pombe*. *J Cell Sci* **114**: 3779–3788.

Huang T, Deng H, Wolkoff AW, Stockert RJ. 2002. Phosphorylation-dependant interaction of the asialoglycoprotein receptor with molecular chaperones. *J Biol Chem* **227**: 37 798–37 803.

Huh WK, Falvo JV, Gerke LC *et al*. 2003. Global analysis of protein localization in budding yeast. *Nature* **425**: 686–691.

Hung DT, Schreiber SL. 1992. cDNA cloning of a human 25 kDa FK506 and rapamycin binding protein. *Biochem Biophys Res Commun* **184**: 733–738.

Hur S, Bruice TC. 2002. The mechanism of *cis-trans* isomerization of prolyl peptides by cyclophilin. *J Am Chem Soc* **124**: 7303–7313.

Inoue T, Yoshida Y, Isaka Y, Tagawa K. 1993. Isolation of mitochondrial cyclophilin from bovine heart. *Biochem Biophys Res Commun* **190**: 857–863.

Ivery M. 2000. Immunophillins: switched-on protein binding domains. *Med Res Rev* **20**: 452–484.

Jin Y, Albers M, Lane W *et al*. 1991. Molecular cloning of a membrane-associated human FK506- and rapamycin-binding protein, FKBP-13. *Proc Natl Acad Sci USA* **88**: 6677–6681.

Jin Y, Burakoff S. 1993. The 25 kDa FK506-binding protein is localized in the nucleus and associates with casein kinase II and nucleolin. *Proc Natl Acad Sci USA* **90**: 7769–7773.

Jin Z-G, Melaragno MG, Liao D-F *et al*. 2000. Cyclophilin A is a secreted growth factor induced by oxidative stress. *Circ Res* **87**: 789–796.

Copyright © 2005 John Wiley & Sons, Ltd. 

Kern G, Kern D, Schmid FX, Fischer G. 1995. A kinetic analysis of the folding of human carbonic anhydrase II and its catalysis by cyclophilin. *J Biol Chem* **270**: 740–745.

Klappa P, Freedman RB, Zimmermann R. 1995. Protein disulphide isomerase and a lumenal cyclophilin-type peptidyl prolyl *cis-trans* isomerase are in transient contact with secretory proteins during late stages of translocation. *Eur J Biochem* **232**: 755–764.

Kops O, Zhou XZ, Lu KP. 2002. Pin1 modulates the dephosphorylation of the RNA polymerase II C-terminal domain by yeast Fcp1. *FEBS Lett* **513**: 305–311.

Koser PL, Bergsma DJ, Cafferkey R *et al.* 1991. The CYP2 gene of *Saccharomyces cerevisiae* encodes a cyclosporin A-sensitive peptidyl-prolyl *cis-trans* isomerase with an N-terminal signal sequence. *Gene* **108**: 73–80.

Krzywicka A, Beisson J, Keller A-M *et al.* 2001. KIN241: a gene involved in cell morphogenesis in *Paramecium tetraurelia* reveals a novel protein family of cyclophilin–RNA interacting proteins (CRIPs) conserved from fission yeast to man. *Mol Microbiol* **42**: 257–267.

Kumar A, Agarwal S, Heyman JA *et al.* 2002. Subcellular localization of the yeast proteome. *Genes Dev* **16**: 707–719.

Kuzuhara T, Horikoshi M. 2004. A nuclear FK506-binding protein is a histone chaperone regulating rDNA silencing. *Nat Struct Mol Biol* **11**: 275–283.

Lai MM, Burnett PE, Wolosker H, Blackshaw S, Snyder SH. 1998. Cain, a novel physiologic protein inhibitor of calcineurin. *J Biol Chem* **273**: 18 325–18 331.

Lam E, Martin MM, Timerman AP *et al.* 1995. A novel FK506 binding protein can mediate the immunosuppressive effects of FK506 and is associated with the cardiac ryanodine receptor. *J Biol Chem* **270**: 26 511–26 522.

Lander ES, Linton LM, Birren B *et al.* 2001. Initial sequencing and analysis of the human genome. *Nature* **409**: 860–921.

Lebeau M, Jung-Testas I, Baulieu E. 1999. Intracellular distribution of a cytoplasmic progesterone receptor mutant and of immunophilins cyclophilin 40 and FKBP59: effects of cyclosporine A, of various metabolic inhibitors and of several culture conditions. *J Steroid Biochem Mol Biol* **70**: 219–228.

Lee S-P, Hwang Y-S, Kim Y-J *et al.* 2001. Cyclophilin A binds to peroxiredoxins and activates their peroxidase activity. *J Biol Chem* **276**: 29 826–29 832.

Lin D-T, Lechleiter JD. 2002. Mitochondrial targeted cyclophilin D protects cells from cell death by peptidyl prolyl isomerization. *J Biol Chem* **277**: 31 134–31 141.

Liu W, Youn H-D, Zhou XZ, Lu KP, Liu JO. 2001. Binding and regulation of the transcription factor NFAT by the peptidyl prolyl *cis-trans* isomerase Pin1. *FEBS Lett* **496**: 105–108.

Lu KP, Hanes SD, Hunter T. 1996. A human peptidyl-prolyl isomerase essential for regulation of mitosis. *Nature* **380**: 544–547.

Lu KP, Liou Y-C, Zhou XZ. 2002. Pinning down proline-directed phosphorylation signaling. *Trends Cell Biol* **12**: 164–172.

Lu PJ, Wulf GG, Zhou XZ, Davies P, Lu K-P. 1999. The prolyl isomerase Pin1 restores the function of Alzheimer's-associated phosphorylated tau protein. *Nature* **399**: 784–788.

Ma D, Nelson LS, LeCoz K, Poole C, Carlow CKS. 2002. A novel cyclophilin from parasitic and free-living nematodes with a unique substrate- and drug-binding domain. *J Biol Chem* **277**: 14 925–14 932.

Maleszka R, Hanes SD, Hackett RL, de Couet HG, Miklos GLG. 1996. The *Drosophila melanogaster* dodo (dod) gene, conserved in humans, is functionally interchangeable with the ESS1 cell division gene of *Saccharomyces cerevisiae*. *Proc Natl Acad Sci USA* **93**: 447–451.

Maleszka R, Lupas A, Hanes SD, Miklos GLG. 1997. The dodo gene family encodes a novel protein involved in signal transduction and protein folding. *Gene* **203**: 89–93.

Manning-Krieg UC, Henriquez R, Cammas F, Graft P, Gaveriaux S, Mowa NR. 1994. Purification of FKBP-70, a novel immunophilin from *Saccharomyces cerevisiae*, and cloning of its structural gene, *FPR3*. *FEBS Lett* **352**: 98–103.

Mark PJ, Ward BK, Kumar P *et al.* 2001. Human cyclophilin 40 is a heat shock protein that exhibits altered intracellular localization following heat shock. *Cell Stress Chap* **6**: 59–70.

Marsh JA, Kalton HM, Gaber RF. 1998. Cns1 is an essential protein associated with the Hsp90 chaperone complex in *Saccharomyces cerevisiae* that can restore cyclophilin 40-dependent functions in Cpr7 deletion cells. *Mol Cell Biol* **18**: 7353–7359.

Maruyama T, Furuani M. 2000. Archaeal peptidyl-prolyl *cis/trans* isomerases (PPIases). *Front Biosci* **5**: 821–836.

Matouschek A, Rospert S, Schmid K, Glick B, Schatz G. 1995. Cyclophilin catalyzes protein folding in yeast mitochondria. *Proc Natl Acad Sci USA* **92**: 6319–6323.

Mayr C, Richter K, Lilie H, Buchner J. 2000. Cpr6 and Cpr7, two closely related HSP90-associated immunophilins from *Saccharomyces cerevisiae*, differ in their functional properties. *J Biol Chem* **275**: 34 140–34 146.

Mazroui R, Pouti A, Kramer A. 1999. Splicing factor SF1 from *Drosophila* and *Caenorhabditis*: presence of an N-terminal RS domain and requirement for viability. *RNA* **5**: 1615–1631.

Messenger MM, Saulnier RB, Gilchrist AD *et al.* 2002. Interactions between protein kinase CK2 and Pin1. Evidence for phosphorylation-dependent interactions. *J Biol Chem* **277**: 23 054–23 064.

Metzner M, Stoller G, Rucknagel KP *et al.* 2001. Functional replacement of the essential ESS1 in yeast by the plant parvulin DlPar13. *J Biol Chem* **276**: 13 524–13 529.

Meunier L, Usherwood Y-K, Chung KT, Hendershot LM. 2002. A subset of chaperones and folding enzymes form multiprotein complexes in endoplasmic reticulum to bind nascent proteins. *Mol Biol Cell* **13**: 4456–4469.

Mi H, Kops O, Zimmermann E, Jaschke A, Tropschug M. 1996. A nuclear RNA-binding cyclophilin in human T cells. *FEBS Lett* **398**: 201–205.

Morris DP, Phatnani HP, Greenleaf AL. 1999. Phospho-carboxyl-terminal domain binding and the role of a prolyl isomerase in pre-mRNA 3′-end formation. *J Biol Chem* **274**: 31 583–31 587.

Munn K, Steward R. 2000. The shut-down gene of *Drosophila melanogaster* encodes a novel FK506-binding protein essential for the formation of germline cysts during oogenesis. *Genetics* **156**: 245–256.

Nagata T, Kishi H, Lui QL *et al.* 2000. Possible involvement of cyclophilin B and caspase-activated deoxyribonuclease in the induction of chromosomal DNA degradation in TCR-stimulated thymocytes. *J Immunol* **165**: 4281–4289.

Nair S, Rimerman R, Toran E *et al.* 1997. Molecular cloning of human FKBP51 and comparisons of immunophilin interactions with Hsp90 and progesterone receptor. *Mol Cell Biol* **17**: 594–603.

Copyright © 2005 John Wiley & Sons, Ltd. 

Nakagawa T, Shimizu S, Watanabe T *et al.* 2005. Cyclophilin D-dependent mitochondrial permeability transition regulates some necrotic but not apoptotic cell death. *Nature* **434**: 652.

Neer EJ, Schmidt CJ, Nambudripad R, Smith TF. 1994. The ancient regulatory-protein family of WD-repeat proteins. *Nature* **371**: 297–300.

Nestel FP, Colwill K, Harper S, Pawson T, Anderson SK. 1996. RS cyclophilins: identification of an NK-TR1-related cyclophilin. *Gene* **180**: 151–155.

Nigam SK, Jin YJ, Jin MJ *et al.* 1993. Localization of the FK506-binding protein, FKBP 13, to the lumen of the endoplasmic reticulum. *Biochem J* **294**: 511–515.

Noguchi N, Takasawa S, Nata K *et al.* 1997. Cyclic ADP-ribose binds to FK506-binding protein 12.6 to release $Ca^{2+}$ from islet microsomes. *J Biol Chem* **272**: 3133–3136.

Obata Y, Yamamoto K, Miyazaki M *et al.* 2005. Role of cyclophilin B in activation of interferon regulatory factor-3. *J Biol Chem* **280**: 18 355–18 360.

Ohi MD, Link AJ, Ren L *et al.* 2002. Proteomics analysis reveals stable multiprotein complexes in both fission and budding yeasts containing Myb-related Cdc5p/Cef1p, novel pre-mRNA splicing factors, and snRNAs. *Mol Cell Biol* **22**: 2011–2024.

Ohi R, Feoktistova A, McCann S *et al.* 1998. Myb-related *Schizosaccharomyces pombe* cdc5p is structurally and functionally conserved in eukaryotes. *Mol Cell Biol* **18**: 4097–4108.

Okadome T, Oeda E, Saitoh M *et al.* 1996. Characterization of the interaction of FKBP12 with the transforming growth factor-beta type I receptor *in vivo*. *J Biol Chem* **271**: 21 687–21 690.

Ozaki K, Fujiwara T, Kawai A *et al.* 1996. Cloning, expression and chromosomal mapping of a novel cyclophilin-related gene (PPIL1) from human fetal brain. *Cytogenet Cell Genet* **72**: 242–245.

Padilla PI, Chang M-J, Pacheco-Rodriguez G *et al.* 2003. Interaction of FK506-binding protein 13 with brefeldin A-inhibited guanine nucleotide-exchange protein 1 (BIG1): effects of FK506. *Proc Natl Acad Sci USA* **100**: 2322–2327.

Page AP, MacNiven K, Hengartner MO. 1996. Cloning and biochemical characterization of the cyclophilin homologues from the free-living nematode *Caenorhabditis elegans*. *J Biochem* **317**: 179–185.

Page AP, Winter AD. 1998. A divergent multi-domain cyclophilin is highly conserved between parasitic and free-living nematode species and is important in larval muscle development. *Mol Biochem Parasitol* **95**: 215–217.

Partaledis J, Berlin V. 1993. The FKB2 gene of *Saccharomyces cerevisiae*, encoding the immunosuppressant-binding protein FKBP-13, is regulated in response to accumulation of unfolded proteins in the endoplasmic reticulum. *Proc Natl Acad Sci USA* **90**: 5450–5454.

Patterson CE, Gao J, Rooney AP, Davis EC. 2002. Genomic organization of mouse and human 65 kDa FK506-binding protein genes and evolution of the FKBP multigene family. *Genomics* **79**: 881–889.

Patterson CE, Schaub T, Coleman EJ, Davis EC. 2000. Developmental regulation of FKBP65. An ER-localized extracellular matrixbinding protein. *Mol Biol Cell* **11**: 3925–3935.

Peattie D, Harding M, Fleming M *et al.* 1992. Expression and characterization of human FKBP52, an immunophilin that associates with the 90 kDa heat shock protein and is a component of steroid receptor complexes. *Proc Natl Acad Sci USA* **89**: 10 974–10 978.

Pemberton TJ, Rulten SL, Kay JE. 2003. Cloning and characterisation of *Schizosaccharomyces pombe* cyclophilin 3: a cyclosporin A-insensitive orthologue of human USA-CyP. *J Chromatogr B* **786**: 81–91.

Pichler A, Gast A, Seeler JS, Dejean A, Melchior F. 2002. The nucleoporin RanBP2 has SUMO1 E3 ligase activity. *Cell* **108**: 109–120.

Picken NC, Eschenlauer S, Taylor P, Page AP, Walkinshaw MD. 2002. Structural and biological characterisation of the gut-associated cyclophilin B isoforms from *Caenorhabditis elegans*. *J Mol Biol* **322**: 15–25.

Pijnappel WWMP, Schaft D, Roguev A *et al.* 2001. The *S. cerevisiae* SET3 complex includes two histone deacetylases, Hos2 and Hst1, and is a meiotic-specific repressor of the sporulation gene program. *Genes Dev* **15**: 2991–3004.

Pratt WB, Toft DO. 1997. Steroid receptor interactions with heat shock protein and immunophillin chaperones. *Endocr Rev* **18**: 306–360.

Price ER, Jin MJ, Lim D *et al.* 1994. Cyclophilin B trafficking through the secretory pathway is altered by binding of cyclosporin A. *Proc Natl Acad Sci USA* **91**: 3931–3935.

Price ER, Zydowsky LD, Jin MJ *et al.* 1991. Human cyclophilin B: a second cyclophilin gene encodes a peptidyl-prolyl isomerase with a signal sequence. *Proc Natl Acad Sci USA* **88**: 1903–1907.

Pringa E, Martinez-Noel G, Muller U, Harbers K. 2001. Interaction of the ring-finger related U-box motif of a nuclear dot protein with ubiquitin-conjugating enzymes. *J Biol Chem* **276**: 19 617–19 623.

Prodromou C, Siligardi G, O'Brien R *et al.* 1999. Regulation of Hsp90 ATPase activity by tetratricopeptide repeat (TPR)-domain co-chaperones. *EMBO J* **18**: 754–762.

Pushkarsky T, Zybarth G, Dubrovsky L *et al.* 2001. CD147 facilitates HIV-1 infection by interacting with virus-associated cyclophilin A. *Proc Natl Acad Sci USA* **98**: 6360–6365.

Ranganathan R, Lu KP, Hunter T, Noel JP. 1997. Structural and functional analysis of the mitotic rotamase Pin1 suggests substrate recognition is phosphorylation dependent. *Cell* **89**: 875–882.

Rassow J, Mohrs K, Koidl S *et al.* 1995. Cyclophilin 20 is involved in mitochondrial protein folding in cooperation with molecular chaperones Hsp70 and Hsp60. *Mol Cell Biol* **15**: 2654–2662.

Reader JS, Van Nuland NAJ, Thompson GS *et al.* 2001. A partially folded intermediate species of the beta-sheet protein apo-pseudoazurin is trapped during proline-limited folding. *Protein Sci* **10**: 1216–1224.

Ren P, Rossettini A, Chaturvedi V, Hanes SD. 2005. The Ess1 prolyl isomerase is dispensable for growth but required for virulence in *Cryptococcus neoformans*. *Microbiology* **151**: 1593–1605.

Riggs DL, Roberts PJ, Chirillo SC *et al.* 2003. The Hsp90-binding peptidylprolyl isomerase FKBP52 potentiates glucocorticoid signaling *in vivo*. *EMBO J* **22**: 1158–1167.

Riviere S, Menez A, Galat A. 1993. On the localization of FKBP25 in T-lymphocytes. *FEBS Lett* **315**: 247–251.

Romano PGN, Horton P, Gray JE. 2004. The *Arabidopsis* cyclophilin gene family. *Plant Physiol* **134**: 1268–1282.

Rubin GM, Yandell MD, Wortman JR *et al.* 2000. Comparative genomics of the eukaryotes. *Science* **287**: 2204–2215.

Copyright © 2005 John Wiley & Sons, Ltd. 

Rulten S, Thorpe J, Kay J. 1999. Identification of eukaryotic parvulin homologues: a new subfamily of peptidylprolyl *cis-trans* isomerases. *Biochem Biophys Res Commun* **259**: 557–562.

Rycyzyn MA, Clevenger CV. 2002. The intracellular prolactin/cyclophilin B complex as a transcriptional inducer. *Proc Natl Acad Sci USA* **99**: 6790–6795.

Rycyzyn MA, Reilly SC, O'Malley K, Clevenger CV. 2000. Role of cyclophilin B in prolactin signal transduction and nuclear retrotranslocation. *Mol Endocrinol* **14**: 1175–1186.

Sanchez E. 1990. Hsp56: a novel heat shock protein associated with untransformed steroid receptor complexes. *J Biol Chem* **265**: 22 067–22 070.

Sanchez E, Ning Y-M. 1996. Immunophilins, heat shock proteins, and glucocorticoid receptor actions *in vivo*. *Comp Methods Enzymol* **9**: 188–200.

Schneuwly S, Shortridge RD, Larrivee DC *et al*. 1989. *Drosophila* ninaA gene encodes an eye-specific cyclophilin (cyclosporine A binding protein). *Proc Natl Acad Sci USA* **86**: 5390–5394.

Schreiber SL, Crabtree GR. 1992. The mechanism of action of cyclosporine A and FK506. *Immunol Today* **13**: 136–142.

Sewell T, Lam E, Martin M *et al*. 1994. Inhibition of calcineurin by a novel FK-506-binding protein. *J Biol Chem* **269**: 21 094–21 102.

Shan X, Xue Z, Melese T. 1994. Yeast NPI46 encodes a novel prolyl *cis-trans* isomerase that is located in the nucleolus. *J Cell Biol* **126**: 853–862.

Shieh BH, Stamnes MA, Seavello S, Harris GL, Zuker CS. 1989. The ninaA gene required for visual transduction in *Drosophila* encodes a homologue of cyclosporin A-binding protein. *Nature* **338**: 67–70.

Shirane M, Nakayama KI. 2003. Inherent calcineurin inhibitor FKBP38 targets Bcl-2 to mitochondria and inhibits apoptosis. *Nat Cell Biol* **5**: 9–11.

Shou W, Aghdasi B, Armstrong DL *et al*. 1998. Cardiac defects and altered ryanodine receptor function in mice lacking FKBP12. *Nature* **391**: 489–492.

Siekierka JJ, Hung SH, Poe M, Lin CS, Sigal NH. 1989. A cytosolic binding protein for the immunosuppressant FK506 has peptidyl-prolyl isomerase activity but is distinct from cyclophilin. *Nature* **341**: 755–757.

Skruzny M, Ambrozkova M, Fukova K *et al*. 2001. Cyclophilins of a novel subfamily interact with SNW/SKIP coregulator in *Dictyostelium discoideum* and *Schizosaccharomyces pombe*. *Biochim Biophys Acta* **1521**: 146–151.

Stamnes MA, Shieh BH, Chuman L, Harris GL, Zuker CS. 1991. The cyclophilin homolog ninaA is a tissue-specific integral membrane protein required for the proper synthesis of a subset of *Drosophila* rhodopsins. *Cell* **65**: 219–227.

Steinmann B, Bruckner P, Superti-Furga A. 1991. Cyclosporin A slows collagen triple-helix formation *in vivo*: indirect evidence for a physiologic role of peptidyl-prolyl *cis-trans* isomerase. *J Biol Chem* **266**: 1299–1303.

Sullivan PG, Thompson MB, Scheff SW. 1999. Cyclosporin A attenuates acute mitochondrial dysfunction following traumatic brain injury. *Exp Neurol* **160**: 226–234.

Syed F, Rycyzyn MA, Westgate L, Clevenger CV. 2003. A novel and functional interaction between cyclophilin A and prolactin receptor. *Endocrine* **20**: 83–90.

Sykes K, Gething M-J, Sambrook J. 1993. Proline isomerases function during heat shock. *Proc Natl Acad Sci USA* **90**: 5853–5857.

Teigelkamp S, Achsel T, Mundt C *et al*. 1998. The 20 kDa protein of human (U4/U6.U5) tri-snRNPs is a novel cyclophilin that forms a complex with the U4/U6-specific 60 kDa and 90 kDa proteins. *RNA* **4**: 127–141.

Tesic M, Marsh JA, Cullinan SB, Gaber RF. 2003. Functional interactions between Hsp90 and the co-chaperones Cns1 and Cpr7 in *Saccharomyces cerevisiae*. *J Biol Chem* **278**: 32 692–32 701.

Theopold U, dal Zotto L, Hultmark D. 1995. FKBP39, a *Drosophila* member of a family of proteins that bind the immunosuppressive drug FK506. *Gene* **156**: 247–251.

Thompson JD, Gibson TJ, Plewniak F, Jeanmougin F, Higgins DG. 1997. The ClustalX windows interface: flexible strategies for multiple sequence alignment aided by quality analysis tools. *Nucleic Acids Res* **24**: 4876–4882.

Timerman AP, Onoue H, Xin H-B *et al*. 1996. Selective binding of FKBP12.6 by the cardiac ryanodine receptor. *J Biol Chem* **271**: 20 385–20 391.

Tropschug M, Nicholson DW, Hartl FU *et al*. 1988. Cyclosporin A-binding protein (cyclophilin) of *Neurospora crassa*. One gene codes for both the cytosolic and mitochondrial forms. *J Biol Chem* **263**: 14 433–14 440.

Uchida T, Fujimori F, Tradler T, Fischer G, Rahfeld JU. 1999. Identification and characterization of a 14 kDa human protein as a novel parvulin-like peptidyl prolyl *cis/trans* isomerase. *FEBS Lett* **446**: 278–282.

Wagenknecht T, Radermacher M, Grassucci R *et al*. 1997. Locations of calmodulin and FK506-binding protein on the three-dimensional architecture of the skeletal muscle ryanodine receptor. *J Biol Chem* **272**: 32 463–32 471.

Waldmeier PC, Feldtrauer J-J, Qian T, Lemasters JJ. 2002. Inhibition of the mitochondrial permeability transition by the nonimmunosuppressive cyclosporin derivative NIM811. *Mol Pharm* **62**: 22–29.

Wang BB, Hayenga KJ, Payan DG, Fisher JM. 1996. Identification of a nuclear-specific cyclophilin which interacts with the proteinase inhibitor eglin c. *Biochem J* **314**: 313–319.

Ward BK, Allan RK, Mok D *et al*. 2002. A structure-based mutational analysis of cyclophilin 40 identifies key residues in the core tetratricopeptide repeat domain that mediate binding to Hsp90. *J Biol Chem* **277**: 40 799–40 809.

Ward BK, Kumar P, Turbett GR *et al*. 2001. Allelic loss of cyclophilin 40, an estrogen receptor-associated immunophilin, in breast carcinomas. *J Cancer Res Clin Oncol* **127**: 109–115.

Warth R, Briand PA, Picard D. 1997. Functional analysis of the yeast 40 kDa cyclophilin Cyp40 and its role for viability and steroid receptor regulation. *Biol Chem* **378**: 381–391.

Weighardt F, Cobianchi F, Cartegni L *et al*. 1999. A novel hnRNP protein (HAP/SAF-B) enters a subset of hnRNP complexes and relocates in nuclear granules in response to heat shock. *J Cell Sci* **112**: 1465–1476.

Weisman R, Creanor J, Fantes P. 1996. A multicopy suppressor of a cell cycle defect in *Sz. pombe* encodes a heat shock inducible 40 kDa cyclophilin-like protein. *EMBO J* **15**: 447–456.

Weisman R, Finkelstein S, Choder M. 2001. Rapamycin blocks sexual development in fission yeast through inhibition of the cellular function of an FKBP12 homolog. *J Biol Chem* **276**: 24 736–24 742.

Wen Y, Shatkin AJ. 1999. Transcription elongation factor hSPT5 stimulates mRNA capping. *Genes Dev* **13**: 1774–1779.

Copyright © 2005 John Wiley & Sons, Ltd. 

Wiederrecht G, Hung S, Chan H *et al*. 1992. Characterization of high molecular weight FK-506 binding activities reveals a novel FK-506-binding protein as well as a protein complex. *J Biol Chem* **267**: 21 753–21 760.

Wilcox CB, Rossettini A, Hanes SD. 2004. Genetic interactions with C-terminal domain (CTD) kinases and the CTD of RNA Pol II suggest a role for ESS1 in transcription initiation and elongation in *Saccharomyces cerevisiae*. *Genetics* **167**: 93–105.

Wood V, Gwilliam R, Rajandream MA *et al*. 2002. The genome sequence of *Schizosaccharomyces pombe*. *Nature* **415**: 871–880.

Wood V, Rutherford KM, Ivens A, Rajandream M-A, Barrell B. 2001. A re-annotation of the *Saccharomyces cerevisiae* genome. *Comp Funct Genom* **2**: 143–154.

Wu J, Matunis MJ, Kraemer D, Blobel G, Coutavas E. 1995. Nup358, a cytoplasmically exposed nucleoporin with peptide repeats, Ran-GTP binding sites, zinc fingers, a cyclophilin A homologous domain, and a leucine-rich region. *J Biol Chem* **270**: 14 209–14 213.

Wu X, Rossettini A, Hanes SD. 2003. The ESS1 prolyl isomerase and its suppressor BYE1 interact with RNA Pol II to inhibit transcription elongation in *Saccharomyces cerevisiae*. *Genetics* **165**: 1687–1702.

Wu X, Wilcox CB, Devasahayam G *et al*. 2000. The Ess1 prolyl isomerase is linked to chromatin remodeling complexes and the general transcription machinery. *EMBO J* **19**: 3727–3738.

Wulf GM, Liou Y-C, Ryo A, Lee SW, Lu KP. 2002. Role of Pin1 in the regulation of p53 stability and p21 transactivation, and cell cycle checkpoints in response to DNA damage. *J Biol Chem* **277**: 47 976–47 979.

Xu YX, Hirose Y, Zhou XZ, Lu KP, Manley JL. 2003. Pin1 modulates the structure and function of human RNA polymerase II. *Genes Dev* **17**: 2765–2776.

Yang W-M, Inouye CJ, Seto E. 1995. Cyclophilin A and FKBP12 interact with YY1 and alter its transcriptional activity. *J Biol Chem* **270**: 15 187–15 193.

Yang W-M, Yao Y-L, Seto E. 2001. The FK506-binding protein 25 functionally associates with histone deacetylases and with transcription factor YY1. *EMBO J* **20**: 4814–4825.

Yao D, Dore JJE Jr, Leof EB. 2000. FKBP12 is a negative regulator of transforming growth factor-beta receptor internalization. *J Biol Chem* **275**: 13 149–13 154.

Young JC, Obermann WMJ, Hartl FU. 1998. Specific binding of tetratricopeptide repeat proteins to the C-terminal 12 kDa domain of hsp90. *J Biol Chem* **273**: 18 007–18 010.

Yu H, Larsen PL. 2001. DAF-16-dependent and -independent expression targets of DAF-2 insulin receptor-like pathway in *Caenorhabditis elegans* include FKBPs. *J Mol Biol* **314**: 1017–1028.

Yurchenko V, O'Connor M, Dai W-W *et al*. 2001. CD147 is a signalling receptor for cyclophilin B. *Biochem Biophys Res Commun* **288**: 786–788.

Yurchenko V, Pushkarsky T, Li J-H *et al*. 2005. Regulation of CD147 cell surface expression: involvement of the proline residue in the CD147 transmembrane domain. *J Biol Chem* **280**: 17 013–17 019.

Yurchenko V, Zybarth G, O'Connor M *et al*. 2002. Active-site residues of cyclophilin A are crucial for its signalling activity via CD147. *J Biol Chem* **277**: 22 959–22 969.

Zacchi P, Gostissa M, Uchida T *et al*. 2002. The prolyl isomerase Pin1 reveals a mechanism to control p53 functions after genotoxic insults. *Nature* **419**: 853–857.

Zaffran S. 2000. Molecular cloning and embryonic expression of dFKBP59, a novel *Drosophila* FK506-binding protein. *Gene* **246**: 103–109.

Zhang J, Herscovitz H. 2003. Nascent lipidated apolipoprotein is transported to the Golgi as an incompletely folded intermediate as probed by its association with network of endoplasmic reticulum molecular chaperones, GRP94, ERp72, BiP, calreticulin, and cyclophilin B. *J Biol Chem* **278**: 7459–7468.

Zheng H, You H, Zhou XZ *et al*. 2002. The prolyl isomerase Pin1 is a regulator of p53 in genotoxic response. *Nature* **419**: 849–853.

Zhou XZ, Kops O, Werner A *et al*. 2000. Pin1-dependent prolyl isomerization regulates dephosphorylation of Cdc25C and tau proteins. *Mol Cell* **6**: 873–883.

Zhou Z, Ying K, Dai J *et al*. 2001. Molecular cloning and characterization of a novel peptidylprolyl isomerase (cyclophilin)-like gene (PPIL3) from human fetal brain. *Cytogenet Cell Genet* **92**: 231–236.

Zorio DA, Blumenthal T. 1999. U2AF25 is encoded by an essential gene clustered in an operon with RRM/cyclophilin in *Caenorhabditis elegans*. *RNA* **5**: 487–494.

Copyright © 2005 John Wiley & Sons, Ltd. 
